# Changed reactivity of secondary hydroxy groups in C8-modified adenosine – lessons learned from silylation

**DOI:** 10.3762/bjoc.16.234

**Published:** 2020-11-23

**Authors:** Jennifer Frommer, Sabine Müller

**Affiliations:** 1 Institute for Biochemistry, University Greifswald, Felix-Hausdorff Str. 4, D-17487 Greifswald, Germany; 2 School of Chemistry, University of Birmingham, Edgbaston, Birmingham B15 2TT, UK

**Keywords:** nucleoside chemistry, protecting groups, RNA synthesis, Sonogashira reaction

## Abstract

Synthesis of site-specifically modified oligonucleotides has become a major tool for RNA structure and function studies. Reporter groups or specific functional entities are required to be attached at a pre-defined site of the oligomer. An attractive strategy is the incorporation of suitably functionalized building blocks that allow post-synthetic conjugation of the desired moiety. A C8-alkynyl-modified adenosine derivative was synthesized, reviving an old synthetic pathway for iodination of purine nucleobases. Silylation of the C8-alkynyl-modified adenosine revealed unexpected selectivity of the two secondary sugar hydroxy groups, with the 3'-*O*-isomer being preferentially formed. Optimization of the protection scheme lead to a new and economic route to the desired C8-alkynylated building block and its incorporation in RNA.

## Introduction

Oligoribonucleotides carrying site-specific modifications are highly required as models for structure and function studies, driven by the ongoing discovery of new RNAs and their investigation [1–6]. This has put demand also on synthetic chemistry to provide suitable compounds at monomeric and oligomeric level. Accordingly, the field has developed to a stage that allows custom-design of RNA probes and tools for specific application. For example, investigations of RNA structures by NMR, EPR, or fluorescence spectroscopy require labeling of the RNA molecules with specific reporter groups [2,4,7–10]. Likewise, assays that implement separation steps require RNA molecules conjugated to an affinity tag such as biotin, or any other functionality for functional selection [11–12]. Very importantly, terminal modification/functionalization is not always suitable to a specific aim. Thus, in addition to building blocks for 5’- or 3’-terminal attachment of a desired functionality, nucleoside derivatives that, upon site-specific incorporation at a pre-determined position of RNA, can be used for post-synthetic conjugation, are required. A number of chemistries are available to specifically attach a molecular entity to RNA in a highly selective and efficient way. The more traditional strategies rely on reaction of isothiocyanates or NHS esters with aliphatic amines [13–14], or on addition of thiols to the α,β-unsaturated carbonyl face of maleimides [15]. Over the past years, the copper catalyzed alkyne–azide cycloaddition (CuAAC) became very popular [16]. A variant of this, the strain-promoted alkyne–azide cycloaddition (SPAAC) even offers the possibility of in cell application, as applies also to the inverse electron-demand Diels–Alder reaction (IEDDA) [17–18]. In vitro, often a combination of orthogonal methods is desired, in order to introduce two or even more functionalities in a specific manner. For example, in earlier work we have used amine-NHS coupling reactions in combination with CuAAC to prepare double labeled RNA molecules for FRET analysis [19]. The conjugation of, sometimes rather large, molecular entities to RNA molecules may disturb functionality, and thus requires careful definition of the conjugation site. As mentioned above, in addition to 5’- and 3’-terminal conjugation, often internal modification of RNA molecules is required. Thus, in order to avoid changes to the RNA sequence, functionalized phosphoramidite building blocks of all four nucleosides are highly desired. The number of commercially available RNA phosphoramidites that carry a suitable functionality for post-synthetic attachment of dyes, reporter groups or other conjugates is still rather limited. In particular, monomer building blocks of the purine nucleosides with functionalities suitable for post-synthetic conjugation at the nucleobase are basically missing, and also in the pyrimidine series, the few existing derivatives of uridine do not offer much variety.

**Scheme 1:**
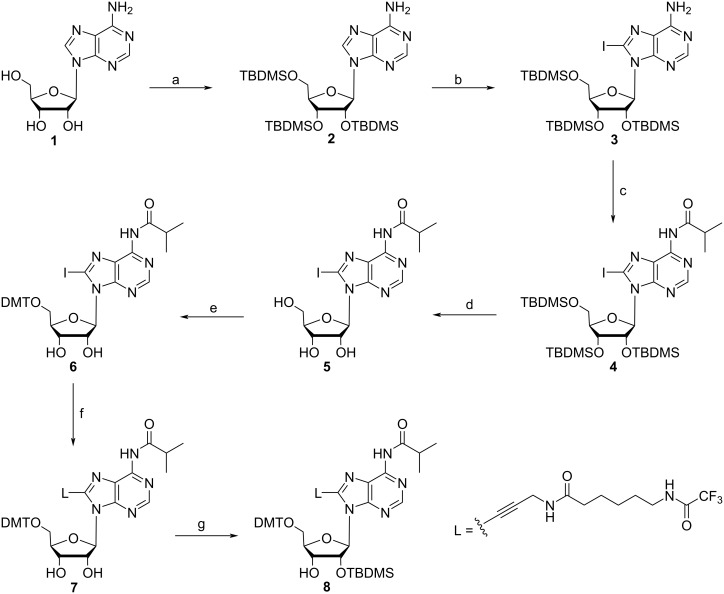
Synthesis of a C8-linker-modified adenosine derivative. (a) 4 equiv TBDMS-Cl, 5 equiv imidazole, DMF, 60 °C, overnight, 82%; (b) 5 equiv LDA, 1.8 equiv I_2_, 5 equiv acetic acid, THF, −75 °C, 9 h, 79%; (c) 6 equiv isobutyric anhydride, pyridine, 45 °C, overnight, 70%; (d) 3.5 equiv TEA·3HF, DMF, room temperature, overnight, 85%; (e) 1.2 equiv DMT-Cl, pyridine, room temperature, 1.5 h, 83%; (f) 0.1 equiv Pd(PPh_3_)_4_, 0.2 equiv CuI, 3 equiv TEA, 1.2 equiv. linker **L**, DMF, room temperature, 19 h, 53%; (g) 1.4 equiv TBDMS-Cl, 1.3 equiv AgNO_3_, 4 equiv pyridine, THF, room temperature, 1.5 h, 10%.

**Scheme 2:**
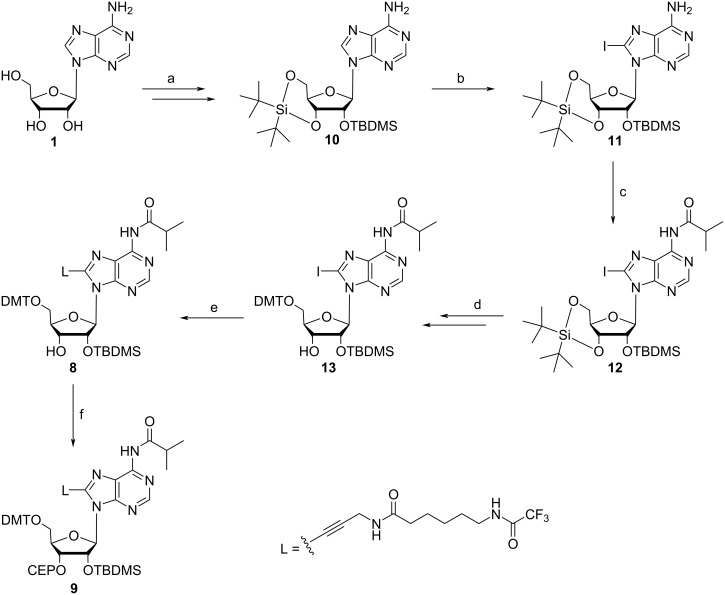
New synthetic route to the C8-linker modified adenosine building block. (a) i) 1.2 equiv di-*tert*-butylsilyl bis(trifluoromethanesulfonate), DMF, 0 °C, 45 min; ii) 5 equiv imidazole, 1.5 equiv TBDMS-Cl, DMF, room temperature, overnight, 83%; (b) 5 equiv LDA, 1.8 equiv I_2_, 5 equiv acetic acid, THF, −75 °C, 9 h, 83%; (c) 6 equiv isobutyric anhydride, pyridine, 45 °C, 24 h, 57%; (d) i) 4 equiv HF (70%) in pyridine, pyridine, DCM, 0 °C, 3 h; ii) 1.3 equiv DMT-Cl, pyridine, room temperature, 1.5 h, 62% over two steps; (e) 0.1 equiv Pd(PPh_3_)_4_, 0.2 equiv CuI, 3 equiv TEA, 1.2 equiv linker L, DMF, room temperature, 24 h, 51%; (f) 1.2 equiv 2-cyanoethyl-*N*,*N*-diisopropylchlorophosphoramidite, 4 equiv TEA, DCM, room temperature, 1 h, 52%.

Motivated by this lack of functional building blocks, we have synthesized a number of pyrimidine and purine derivatives carrying amino linkers of different length and flexibility [13,20]. Linker-modified uridine derivatives, upon conversion into phosphoramidite building blocks, were incorporated in RNA and used for a systematic study of distance determination of nucleic acids via Förster Resonance Energy Transfer (FRET) [20]. More recently, we started an effort to develop an efficient strategy for the preparation of a linker-modified adenosine building block, which in a future project is to be used for post synthetic conjugation of reporters or functional entities in our ribozyme design projects [21–22]. Strikingly, the C8-position of a specific adenosine in the loop region of the flavine mononucleotide (FMN) aptamer is a highly favorable position for covalent attachment of FMN to study regulation of an FMN dependent hairpin aptazyme in response to RNA charge transfer [23–24]. In the course of monomer synthesis, we encountered unexpected results regarding the reactivity and selectivity of the two secondary hydroxy groups of the adenosine derivative **7** (Scheme 1) in the silylation step, leading to non-satisfactory overall reaction yields. Therefore, the synthesis strategy was re-designed, allowing the preparation of building block **9** (Scheme 2) ready for use in solid-phase RNA synthesis with excellent yield. Here, we report on the selectivity problem in 2’-*O-*silylation of adenosine derivative **7** (Scheme 1) and the optimized synthesis strategy for the phosphoramidite building block **9** (Scheme 2).

## Results and Discussion

Typically, the synthesis of C8-alkynyl derivatives relies on C8-bromoadenosine as reactant for the Sonogashira cross-coupling reaction to introduce the amino linker *N*-(propyn-2-yl)-6-(trifluoroacetamido)hexanamide (**L**) bearing an alkynyl moiety [25]. Therefore, we decided to start the synthesis with the preparation of the C8-brominated derivative. Halogenation with bromine was achieved in good yields, however, the following Sonogashira reaction reproducibly proceeded with very low yields (data not shown). Therefore, we changed the used halide to iodine, taking into account that direct iodination of purines has been claimed being troublesome [26], although not impossible [27]. For C8-iodination of adenosine, first the hydroxy groups at the sugar moiety were protected with *tert*-butyldimethylsilyl (TBDMS) groups. The silylated nucleoside **2** was dissolved in THF and lithium diisopropylamide (LDA) was added, followed by iodine in THF. The reaction temperature was kept strictly between −70 and −80 °C to make sure that iodination proceeds without further side reactions (Scheme 1) [28]. Despite the fact that the exocyclic amino group was not protected, side reactions were not observed and good yields (79%) of the C8-iodo derivative **3** were achieved.

Prior to Sonogashira coupling of the linker moiety, the exocyclic amine of the nucleoside derivative was protected with an isobutyryl group, and the silyl groups at the sugar hydroxy functions were removed. We used TEA·3HF in DMF for this purpose, allowing easy purification of the deprotected nucleoside derivative **5** by crystallization from DCM with a yield of 60% over two reaction steps.

**Figure 1:**
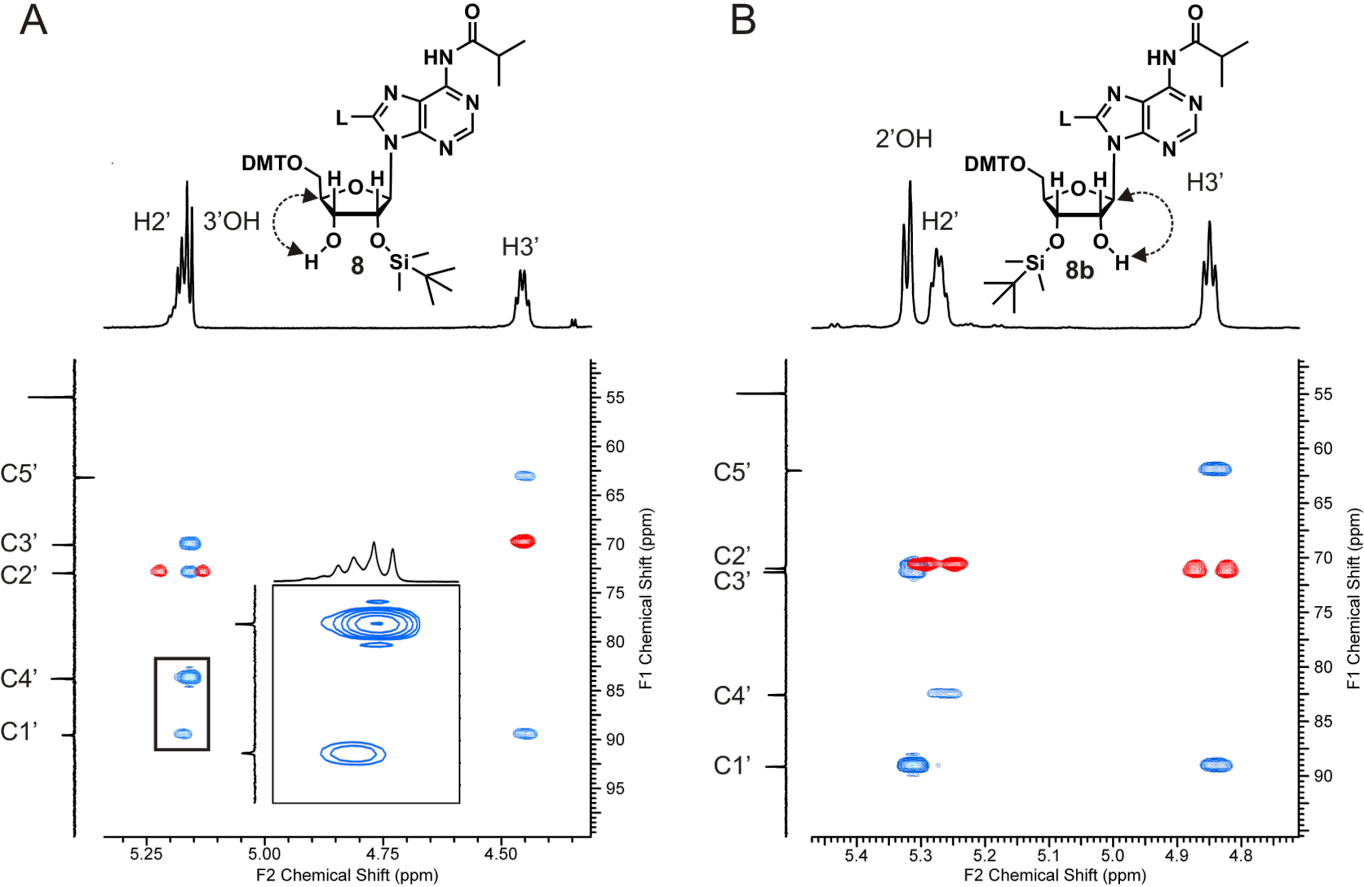
Characterization and assignment of the TBDMS isomers via HSQC (red) and HMBC (blue) NMR measurements. A) The merged ^1^H signal at 5.17 ppm results from the H2’ and an OH group, since the H3’ can be clearly identified through the HMBC correlation with C5’. The zoomed region shows the HMBC correlation of the OH group with C4’, which together with the weaker signal between H2’ and C1’ identifies this nucleoside as the 2’-*O*-TBDMS isomer. B) The H3’-signal is distinct from the H3’ signal in A in its multiplicity, and the OH group has a HMBC correlation with C1’, not with C4’, which identifies this nucleoside as the 3’-*O*-TBDMS isomer.

Next, the 5’-hydroxy group was protected with DMT, and the linker on C8 was introduced by Sonogashira coupling following a previously established protocol [13], resulting in nucleoside derivative **7** with 44 % yield over these two steps, corresponding to an overall yield of 17% over six reaction steps. Further functionalization of **7** for RNA synthesis required selective 2’-*O*-silylation to deliver derivative **8** with free 3’-OH group, which then can be converted to the phosphoramidite prior to use at the RNA synthesizer. Protocols for selective 2’-*O*-silylation are available [29–31], however, the standard procedure using AgNO_3_, pyridine and TBDMS-Cl, in this case led to unexpected results. The reaction was monitored by TLC, whereby two product spots were observed, though the product with the lower *R*_f_ value seemed to have formed preferentially. This was confirmed after separation of both products via column chromatography, the ratio of the product with the higher *R*_f_ value to the one with the lower *R*_f_ value was 1:4. In general, the 2’-*O*-isomer tends to have a higher *R*_f_ value than the 3’-*O-*isomer [31], which would mean that with the linker-modified adenosine derivative **8**, preferentially the 3’-*O*-isomer has formed under standard conditions of the silylation procedure. For clarification, both isomers were characterized via HSQC and HMBC NMR spectroscopy (Figure 1).

The ^13^C and ^1^H measurements of the two regioisomers show remarkable differences in chemical shifts and multiplicity of the relevant signals, though the merged signal of H2’ and an OH group in one of the spectra (Figure 1A) impedes the evaluation. The H3’-signal was easily assigned owing to its HMBC correlation with C5’, which is missing for all of the other protons. Its distinct multiplicity in the two spectra in Figure 1 already indicates a different coupling environment in the two isomers. The merged signal of H2’ and 3’-OH in Figure 1A reveals a HMBC correlation of both protons with C4’, but only H2’ shows a correlation with C1’. In Figure 1B, the OH signal shows a correlation with C1’, and, very importantly, not with C4’, which in combination with the distinct multiplicity of H3’ clearly indicates that the spectrum in Figure 1A corresponds to the 2’-*O*-silylated isomer, and the spectrum in Figure 1B to the 3’-*O*-silylated isomer. Hence, in the TLC analysis done before, the spot with the higher *R*_f_ value represents the desired 2’-*O*-TBDMS isomer in agreement with what is said in the literature [31]. However, the 2’-/3’-*O*-silylated isomer ratio is 1:4, and thus indicates that the 3’-*O*-silyl isomer has formed preferentially, even though the recommended conditions for preferred silylation of the 2’-OH position were chosen [29–31]. According to the literature and to our experience over years, AgNO_3_ is the important additive that decides on preferential 2’-*O*-silylation. The salt has been suggested to influence reaction kinetics in the way that the silylation reagent TBDMS chloride is changed to the nitrate, which subsequently is consumed faster by nucleophilic attack of the 2’-OH group onto the silica atom as compared with the 3’-OH group, due its higher acidity [29]. For modified nucleosides, the preference of 2’-*O*-TBDMS formation in the presence of AgNO_3_ may not be given [29], and indeed, as already mentioned above, the C8-linker conjugated nucleoside derivative **7** (Scheme 1) shows the opposite behavior: the 3’-*O*-TBDMS isomer has formed preferentially. Therefore, we decided to let the reaction proceed in the absence of AgNO_3_, conditions that have been supposed to deliver both isomers in equal amount. In addition, the amount of the silylation reagent, the solvent, the nature of the catalyst and the base as well as the temperature were varied in order to find conditions for preferred 2’-*O*-silylation (Table 1). Unfortunately, all tested reaction conditions failed. AgNO_3_ was found being absolutely essential for the reaction to proceed. In its absence neither the 2’-, nor the 3’-isomer was formed, whereas in the presence of AgNO_3_ the 3’-*O*-TBDMS derivative was always obtained in excess over the 2’-isomer.

**Table 1:** Variation of reaction conditions for 2’-/3’-*O* silylation of adenosine derivative **7**.

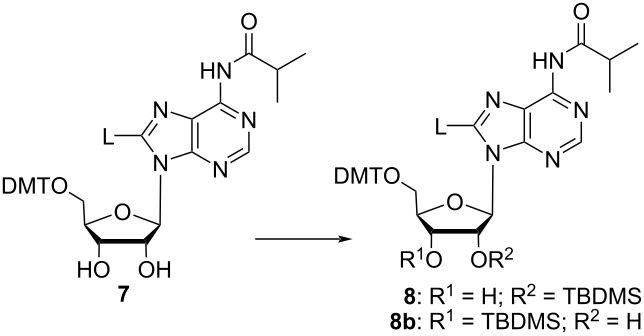							

Entry	TBDMSCl	Catalyst	Base	*T* (°C)	Solvent	Yield **8**	Yield **8b**

1	1.4 equiv	AgNO_3_	pyridine	rt	THF	10%	40%
2	1.3 equiv	–	pyridine	rt	THF	–	–
3	1.5 → 5 equiv	–	imidazole	40	DMF	–	–
4	1.3 equiv	DMAP	pyridine	rt	THF	–	–
5	1.1 equiv	DMAP + AgNO_3_	pyridine	rt	THF	10%	40%

The differences in the reactivity of the 2’- and 3’-OH groups mirror the impact of the sugar conformation, which is dependent on the substitution pattern. Ribonucleosides favor the 3’-endo conformation (or type N-conformers) [32], and the normally observed higher reactivity of the 2’-OH group in silylation reactions can be correlated with it. A closer examination of the coupling constants of the sugar protons revealed a shift from 6.3 Hz to 4.5 Hz for *J*_1’-2’_ and a minor shift from 4.9 Hz to 5.5 Hz for *J*_2’-3’_ after 5'-O-dimethoxytritylation of compound **5** to obtain derivative **6**. The clear shift of *J*_1’-2’_ can be taken as evidence for a changed sugar conformation, supported by the *J*_1’-2’_ coupling constant of 6.1 Hz found in the literature for the 3’-endo conformation of adenosine [32]. Introduction of the linker **L** at C8 in derivative **7** changes the *J*_1’-2’_ coupling constant only slightly (4.8 Hz *J*_1’-2’_), however, brings in a strong steric effect and most likely induces a preferential *syn*-conformation of the nucleobase relative to the sugar residue. These effects together can be accounted for the observed reactivity changes, favoring the 3'-OH group of derivative **7** as silylation site.

Conversion of the 3’-*O*-silyl isomer to the 2’-*O*-silyl isomer can be accomplished by solvation of the 3*’*-isomer in methanol under slightly basic conditions, such that nucleophilic attack of the 2’-OH onto the neighboring silicon leads to silyl migration and consequently to an isomeric mixture, which can be separated by column chromatography [30]. However, this procedure does not secure high yields and the careful separation of a sufficient amount of the 2’-*O*-silyl isomers is rather time consuming. Additionally, the evaluation of a fast and high yield synthetic route for obtaining the modified ribonucleoside building block is highly desirable. For this reason, we have redesigned the synthesis strategy, and decided to use di-*tert-*butylsilyl bis(trifluoromethanesulfonate) as reagent for 3’,5’-di-*O*-protection of adenosine [33–34]. The 3′,5′-*O*-di-*tert-*butylsilyl protecting group, in contrast to the Markiewicz group (1,1,3,3-tetraisopropyldisiloxane) can be selectively removed with HF-pyridine [35–36]. It was used for the iodination of cytosine residues previously [37], but to the best of our knowledge never for the iodination of a purine nucleobase, which is achieved under harsher conditions. Thus, the 3′,5′-*O*-di-*tert-*butylsilyl protecting group was introduced, followed by reaction of the 2’-OH group with TBDMS chloride to generate intermediate **10** (Scheme 2). Subsequently, the iodination was carried out without changing the reaction conditions used for the previous iodination of **2**, resulting in the product **11** with a yield of 83%. The protection of the exocyclic amine lead to nucleoside intermediate **12**, from which the 3′,5′-*O*-di-*tert-*butylsilyl group was selectively removed with HF-pyridine without harming the 2’-*O*-TBDMS ether [35,38–39]. Subsequently, the 5’-OH group was protected with DMT, and the resulting adenosine derivative was reacted with the amino linker **L** under Sonogashira conditions to obtain the nucleoside linker conjugate **8**. Final 3’-*O*-phosphitylation yielded the phosphoramidite building block **9** ready for use in solid-phase RNA synthesis.

When starting the synthesis via this way, we were not sure, if the protected adenosine derivative **10** is a suitable substrate for iodination. The cyclic nature of the 3′,5′-*O*-di-*tert-*butylsilyl group is associated with a slight ring strain energy, which allows its selective removal with simultaneous preservation of the 2’-*O*-TBDMS group. This advantage on the one hand, might cause problems on the other. It was not for sure, if the cyclic silyl ether would be sufficiently stable under the conditions of iodination and Sonogashira cross coupling, and even if so, how it would influence both reaction steps in terms of reactivity and product yield. To our satisfaction, iodination of **10** proceeded smoothly with 83% yield, and also the following Sonogashira reaction delivered the nucleoside linker conjugate **8** in moderate yield (51%). We observed partial migration of the TBDMS protecting group and consequently formation of the 3’-*O*-TBDMS isomer under Sonogashira conditions, which accounts for the reduced yield. This certainly can be counteracted by further reducing the reaction temperature of the Sonogashira coupling. Under the conditions applied here, formation of the desired adenosine derivative **8** was achieved with an overall yield of 12.4% over seven reaction steps. A mentioned above, 3’-*O*-phosphitylation of **8** was carried out [40], and the resulting phosphoramidite building block **9** was used for the synthesis of an oligoribonucleotide (Table S1 in Supporting Information File 1). The presence of the modified ribonucleotide in the synthesized sequence was confirmed by MALDI–TOF MS (Figure S1, Supporting Information File 1).

## Conclusion

Oligonucleotides carrying a specific modification or functional entity at a pre-defined position are in high demand for structure and function studies of nucleic acids. Often, the effort to synthesize a specifically modified oligonucleotide is underestimated, since a wide spectrum of precursors and standard methodology is available. However, dependent on the specific synthetic aim, standard methods can fail or lead to unexpected results, making thoughtful design of the synthetic route on one hand, and careful analysis of products on the other necessary. The introduction of a TBDMS group to the 2’-OH functionality of a ribonucleotide routinely proceeds by reaction of the 5’-*O*-DMT-*N*-acyl protected nucleoside with TBDMS-Cl in the presence of AgNO_3_, yielding a mixture of two regioisomers, although with the 2’-*O*-TBDMS protected isomer in excess over the 3’-*O*-TBDMS isomer [31]. Both species can be separated by chromatography; and often it is trusted that the isomer with the higher *R*_f_ value is the desired 2’-*O*-isomer. As concluded from NMR analysis, this also applies to the adenosine derivative reported here. However, standard reaction conditions that should preferentially lead to the 2’-*O*-TBDMS isomer, here favored formation of the 3’-*O*-isomer in fourfold excess, and we were not able to find conditions that would reverse this ratio. Therefore, a different synthetic route was chosen, using a cyclic silyl group for bridged protection of the 3’-, and 5’-OH groups, allowing reaction of the remaining free 2’-OH group with TBDMS-Cl, followed by selective removal of the 3’,5’-protecting group. This is a clear advantage over the traditional method of reacting the 2’-, 3’- unprotected nucleoside and subsequently separating the formed regioisomers, in particular if like here, the desired 2’-*O*-isomer is the minor product. Moreover, the regiospecific iodination of the C8-position of 3’,5’-*O*-di*-tert-*butylsilyl-2’-*O*-TBDMS protected adenosine derivative **10** could be achieved with high yields, demonstrating that the cyclic protecting group does not hinder selective iodination at the purine nucleobase. This route enabled us to obtain derivative **13** with high yields (24% in 6 steps); ready to be used as a universal reactant for various Pd-catalyzed reactions. Comparing the two reaction paths, the overall yield of the desired adenosine derivative **8** was increased from 2% to 12% over 7 reaction steps by changing the protecting group strategy.

## Supporting Information

File 1 Experimental procedures, RNA synthesis, characterization data (^1^H, ^13^C NMR, MALDI–TOF MS, PAGE), copies of ^1^H and ^13^C NMR spectra.
