# Supplementary material for: Changed reactivity of secondary hydroxy groups in C8-modified adenosine – lessons learned from silylation
Source: Beilstein J Org Chem. 2020 Nov 23;16:2854–61. doi: 10.3762/bjoc.16.234 (PMC7705864; doi:10.3762/bjoc.16.234)
Supplement: File 1 — Experimental procedures, RNA synthesis, characterization data (1H, 13C NMR, MALDI–TOF MS, PAGE), copies of 1H and 13C NMR spectra. [file Beilstein_J_Org_Chem-16-2854-s001.pdf]

BEILSTEIN JOURNAL OF ORGANIC CHEMISTRY

Supporting Information

for

Changed reactivity of secondary hydroxy groups in C8-modified adenosine – lessons learned from silylation

Jennifer Frommer and Sabine Müller

Beilstein J. Org. Chem. **2020,** *16,* 2854–2861. doi:10.3762/bjoc.16.234

Experimental procedures, RNA synthesis, characterization data (^{1}H, ^{13}C NMR, MALDI–TOF MS, PAGE), copies of ^{1}H and ^{13}C NMR spectra

License and Terms: This is a supporting information file under the terms of the Creative Commons Attribution License (https://creativecommons.org/licenses/by/4.0). Please note that the reuse, redistribution and reproduction in particular requires that the authors and source are credited.
The license is subject to the *Beilstein Journal of Organic Chemistry* terms and conditions: (https://www.beilstein-journals.org/bjoc)

Experimental section

General

Triethylamine (TEA) was stored over calcium hydride, pyridine over KOH; both solvents were freshly distilled before use. All other reagents, chemicals and solvents were obtained as the highest commercially available grade and used without further purification. All reactions were carried out in dry solvents under argon atmosphere. For the Sonogashira reactions the solvents were additionally degassed using an argon stream. Silica gel for column chromatography (0.063–0.2 mm) was obtained from Merck. TLC chromatography was performed on pre-coated aluminium silica gel 60 F254 plates (Merck). NMR spectra (^{1}H, ^{13}C, ^{31}P, HMBC, HSQC) were acquired on a Bruker Avance 600 MHz spectrometer, assignment of the NMR signals was carried out by 2D NMR measurements (HMBC, HSQC). Mass spectra were recorded on a Bruker microflex MALDI–TOF MS. Standard PAC-phosphoramidites as well as CPG supports were obtained from ChemGenes or Link Technologies. The oligoribonucleotide was synthesized on a Pharmacia LKB Gene Assembler 4 Primers DNA/RNA Synthesizer following our standard protocol for oligoribonucleotide chain assembly [1]. According to the detritylation assay, the adenosine derivative **9** was coupled with 90% efficiency. For removal of base and phosphate protecting groups and cleavage from the support the synthesized RNAs were incubated with aqueous ammonia (32%)/ethanolic methylamine (8 M) (1:1, v/v) at 65 °C for 40 min. Afterwards all RNAs were incubated with TEA·3HF for 1.5 h at 55 °C for removal of the 2'-*O*-protecting groups.

Caution, the here described syntheses were carried out under the usage of triethylamine trihydrofluoride TEA·3HF (H300 + H310 + H330 - H314 and 70% HF pyridine solution (H225-H300 + H310 + H330-H314). Both HF solutions should be handled with care and the right Personal Protective Equipment should be provided.

Table S1 Mass data of the synthesized RNA sequence

	**X** = **8LA**		
	sequence	m/z $[M+H]^+$	calc. $[M+H]^+$
**RNA1**	5’ GGC GUG UAG G**X**U AUG CCC 3’	5956	5954
		m/z $[M+2H]^{2+}$	calc. $[M+2H]^{2+}$
		2976	2977

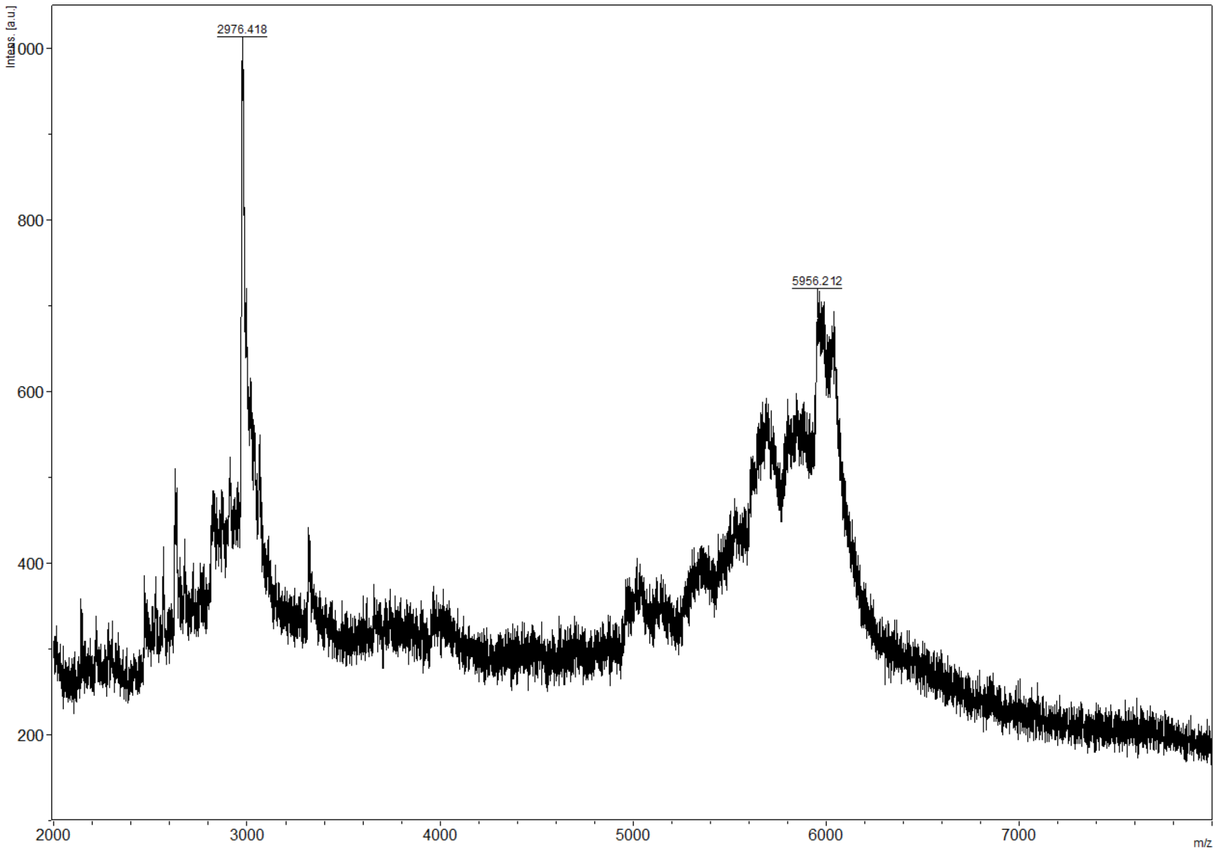

Figure S1 MALDI–TOF spectra of the synthesized RNA1.

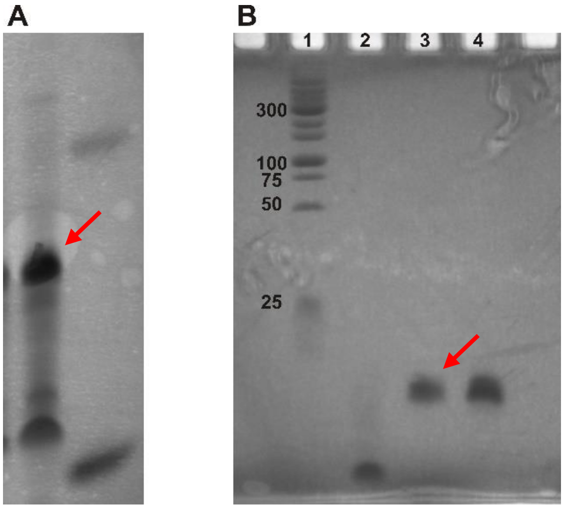

Figure S2 A) 15% den. PAGE of the RNA1 (red arrow) crude product. B) 15% den. PAGE of a DNA ladder (lane 1), 7mer RNA reference (lane 2), RNA1 (red arrow, lane 3), 18mer RNA reference (lane 4).

2´,3´,5´-Tris-*O*-(*tert*-butyldimethylsilyl)-adenosine (2)

Synthesis of compound **2** was described before [2].

Adenosine (5 g, 18.7 mmol) was dried by evaporation with dry DCM three times, purged with argon and dissolved in 50 ml dry DMF, followed by the addition of imidazole (6.4, 94 mmol) and TBDMS-Cl (11.3 g, 75 mmol). The reaction mixture was stirred overnight at 60 °C and afterwards the DMF was removed under reduced pressure. The residue was resolved in DCM, the resulting solution was washed twice with saturated aq. $NaHCO_3$, and the combined aq. phase was extracted with DCM. The combined organic phase was dried with Na_2SO_4 and evaporated to dryness. After column chromatography (DCM/MeOH 99:1 to 95:5) compound **2** was obtained as a colourless solid (9.4 g, 15.4 mmol, 82%).

R_f 0.50 (‘DCM/MeOH 95:5), ^{1}H-NMR (600 MHz, DMSO-d_6): δ 8.33, 8.12 (2H, s, s, H2/H8) 7.31 (2H, br s, NH_2) 5.93 (1H, d, *J* 6.4 Hz, H1‘) 4.91 (1H, dd, *J* 6.4 Hz, H2’) 4.32 (1H, m, H3’) 4.00 (2H, m, H4‘/H5‘(a)) 3.73 (1H, dd, *J* 7.1 Hz, H5‘(b)) 0.92 (9H, s, $SiC(CH_3)_3$) 0.89 (9H, s, $SiC(CH_3)_3$) 0.71 (9H, s, $SiC(CH_3)_3$) 0.13 (3H, s, $SiCH_3$) 0.11 (3H, s, $SiCH_3$) 0.08 (3H, s, $SiCH_3$) 0.07 (3H, s, $SiCH_3$) -0.11 (3H, s, $SiCH_3$) -0.36 (3H, s, $SiCH_3$) ^{13}C-NMR (150 MHz, DMSO-d_6): δ 156.10 (q, C4) 152.56 (CH, C2) 149.41 (q, C6) 139.47 (CH, C8) 119.17 (q, C5) 86.81 (CH, C1’) 85.23 (CH, C4’) 74.21 (CH, C2’) 72.34 (CH, C3’) 62.46 (CH_2, C5’) 25.79 (CH_3, $SiC(CH_3)_3$) 25.71 (CH_3, $SiC(CH_3)_3$) 25.45 (CH_3, $SiC(CH_3)_3$) 18.01 (q, $SiC(CH_3)_3$) 17.78 (q, $SiC(CH_3)_3$) 17.49 (q, $SiC(CH_3)_3$) -4.69 ($SiCH_3$) -4.84 ($SiCH_3$) -4.88 ($SiCH_3$) -5.48 ($SiCH_3$) -5.49 ($SiCH_3$) -5.56 ($SiCH_3$), MALDI-TOF *m/z* 610.76 ($[M + H]^+$, $C_{28}H_{55}N_5O_4Si_3H^+$ calc. 610.36)

2´,3´,5´-Tris-*O*-(*tert*-butyldimethylsilyl)-8-iodoadenosine (3)

2´,3´,5´-Tris-*O*-(*tert*-butyldimethylsilyl)-adenosine (**2**, 8 g, 13.1 mmol) was dried by evaporation with dry DCM three times, purged with argon and then dissolved in 74 ml dry THF in a three-neck flask. The solution was cooled down at −80 °C in an isopropanol bath. A 2 M LDA solution (33 ml, 66 mmol, solution in THF, heptane and ethylbenzene) was diluted under argon in 60 ml dry THF and added to the nucleoside solution via a dropping funnel. The temperature of the reaction mixture was monitored and did not exceed a temperature of -70 °C. Afterwards the reaction mixture was stirred for 5.5 h at −75 °C. In the meantime, iodine (6 g, 23.6 mmol) was dissolved in 50 ml dry THF under argon and added to the reaction mixture with a dropping funnel, whereas the temperature did not exceed a temperature of −70 °C. The reaction mixture was stirred for additional 3 h at −75 °C before the isopropanol bath was removed and the reaction mixture was allowed to reach room temperature. The reaction was stopped by addition of acetic acid (3.75 ml, 66 mmol), followed by dilution in 1 l ethyl acetate. The resulting solution was washed with water until the water phase remained clear and colourless. The organic phase was dried with Na_2SO_4 and evaporated to dryness. After column chromatography (hexane/ethyl acetate 3:1 to 1:1) compound **3** was obtained as a white solid (7.6 g, 10.3 mmol, 79%).

R_f 0.45 (hexane/ethyl acetate 1:1), ^{1}H-NMR (600 MHz, DMSO-d_6): δ 8.04 (1H, s, H2) 7.43 (2H, br s, NH_2) 5.81 (1H, d, *J* 6.1 Hz, H1') 5.59 (1H, dd, *J* 5.9 Hz, H2') 4.54 (1H, m, H3') 4.08 (1H, dd, *J* 10.9 Hz, H4') 3.95 (1H, m, H5'(a)) 3.67 (1H, dd, *J* 11.0 Hz, H5'(b)) 0.94 (9H, s, $SiC(CH_3)_3$) 0.81 (9H, s, $SiC(CH_3)_3$) 0.76 (9H, s, $SiC(CH_3)_3$) 0.15 (3H, s, $SiCH_3$) 0.14 (3H, s, $SiCH_3$) -0.01 (3H, s, $SiCH_3$) -0.05 (3H, s, $SiCH_3$) -0.11 (3H, s, $SiCH_3$) -0.40 (3H, s, $SiCH_3$) ^{13}C-NMR (150 MHz, DMSO-d_6): δ 155.03 (q, C4) 151.86 (CH, C2) 149.40 (q, C6) 122.45 (CH, C5) 104.07 (q, C8) 91.33 (CH, C1') 87.67 (CH, C4') 73.07 (CH, C2') 71.79 (CH, C3') 61.68 (CH_2, C5') 25.81 (CH_3, $SiC(CH_3)_3$) 25.72 (CH_3, $SiC(CH_3)_3$) 25.56 (CH_3, $SiC(CH_3)_3$) 17.80 (q, $SiC(CH_3)_3$) 17.75 (q, $SiC(CH_3)_3$) 17.48 (q, $SiC(CH_3)_3$) -3.19 ($SiCH_3$) -4.60 ($SiCH_3$) -4.85 ($SiCH_3$) -4.95 ($SiCH_3$) -5.78 ($SiCH_3$) -5.82 ($SiCH_3$), MALDI-TOF *m/z* 735.95 ([M + H]$^+$, $C_{28}H_{54}IN_5O_4Si_3H^+$ calc. 736.25)

2´,3´,5´-Tris-*O*-(*tert*-butyldimethylsilyl)-6-*N*-isobutyryl-8-iodoadenosine (4)

2´,3´,5´-Tris-*O*-(*tert*-butyldimethylsilyl)-8-iodoadenosine (**3**, 2.5 g, 3.4 mmol) was dried by evaporation with dry pyridine three times, purged with argon and was dissolved in 17 ml dry pyridine, followed by the addition of isobutyric anhydride (3.3 ml, 20.4 mmol) under argon. The reaction mixture was stirred over night at 45 °C. Afterwards the pyridine was removed under reduced pressure and the residue was twice coevaporated with toluene and three times with ethanol. The residue was resolved in ethyl acetate and the resulting solution was washed twice with saturated aq. $NaHCO_3$. The combined aq. phase was extracted with ethyl acetate. The combined organic phase was dried with Na_2SO_4 and evaporated to dryness. After column chromatography (hexane/ethyl acetate 9:1 to 8:2) compound **4** was obtained as a white solid (1.9 g, 2.4 mmol, 70%).

R_f 0.45 (hexane/ethyl acetate 8:2), ^{1}H-NMR (600 MHz, DMSO-d_6): δ 10.76 (1H, s, NH) 8.55 (1H, s, H2) 5.89 (1H, d, *J* 5.8 Hz, H1') 5.55 (1H, dd, *J* 5.3 Hz, H2') 4.59 (1H, m, H3') 4.03 (1H, m, H4') 3.98 (1H, m, H5'(a)) 3.71 (1H, m, H5'(b)) 2.94 (1H, sept, *J* 6.8 Hz, C**H**$(CH_3)_2$) 1.12 (6H, d, *J* 6.8 Hz, $CH(CH_3)_2$) 0.95 (9H, s, $SiC(CH_3)_3$) 0.79 (9H, s, $SiC(CH_3)_3$) 0.76 (9H, s, $SiC(CH_3)_3$) 0.16 (3H, s, $SiCH_3$) 0.15 (3H, s, $SiCH_3$) -0.01 (3H, s, $SiCH_3$) -0.08 (3H, s, $SiCH_3$) -0.09 (3H, s, $SiCH_3$) -0.41 (3H, s, $SiCH_3$) ^{13}C-NMR (150 MHz, DMSO-d_6): δ 175.21 (q, C=O) 151.99 (q, C4) 151.11 (CH, C2) 148.73 (q, C6) 126.55 (q, C5) 110.43 (q, C8) 91.69 (CH, C1') 84.90 (CH, C4') 71.66 (CH, C2') 71.08 (CH, C3') 61.69 (CH_2, C5') 34.39 (CH, $CH(CH_3)_2$) 25.73 (CH_3, $SiC(CH_3)_3$) 25.59 (CH_3, $SiC(CH_3)_3$) 25.49 (CH_3, $SiC(CH_3)_3$) 19.24 (CH_3, $CH(CH_3)_2$) 19.19 (CH_3, $CH(CH_3)_2$) 17.89 (q, $SiC(CH_3)_3$) 17.79 (q, $SiC(CH_3)_3$) 17.51 (q, $SiC(CH_3)_3$) -4.71 ($SiCH_3$) -4.73 ($SiCH_3$) -4.81 ($SiCH_3$) -5.41 ($SiCH_3$) -5.49 ($SiCH_3$) -5.66 ($SiCH_3$), MALDI-TOF *m/z* 805.89 ([M + H]$^+$, $C_{32}H_{60}IN_5O_5Si_3H^+$ calc. 806.29)

6-*N*-Isobutyryl-8-iodoadenosine (5)

2´,3´,5´-Tris-*O*-(*tert*-butyldimethylsilyl)-6-*N*-isobutyryl-8-iodoadenosine (**4**, 5 g, 18.7 mmol) was dissolved in 19 ml dry DMF, followed by the addition of $Et_3N{\cdot}3HF$ (10.7 ml, 65.5 mmol). The resulting solution was stirred over night at room temperature and afterwards the DMF was removed under reduced pressure. The residue was resolved in DCM and the product

precipitated. The precipitate was separated from the solvent via a Büchner funnel, and compound **5** was obtained as a white solid (0.9 g, 2 mmol, 85%).

R_f 0.28 (DCM/MeOH 95:5), ^{1}H-NMR (600 MHz, DMSO-d_6): δ 10.76 (1H, s, NH) 8.58 (1H, s, H2) 5.85 (1H, d, *J* 6.3 Hz, H1‘) 5.48 (1H, d, *J* 6.2 Hz, 2‘OH) 5.29 (1H, d *J* 4.8 Hz, 3‘OH) 5.23 (1H, dd, *J* 6.1 Hz, H2’) 5.07 (1H, dd, *J* 4.9 and 7.4 Hz, 5’OH) 4.26 (1H, dd, *J* 4.9 and 7.9 Hz, H3’) 3.96 (1H, dd, *J* 4.7 and 7.9 Hz, H4‘) 3.69 (1H, m, H5‘(a)) 3.53 (1H, m, H5‘(b)) 2.94 (1H, sept, *J* 6.8 Hz, C**H**$(CH_3)_2$) 1.12 (6H, d, *J* 6.8 Hz, $CH(CH_3)_2$) ^{13}C-NMR (150 MHz, DMSO-d_6): δ 175.26 (q, C=O) 152.07 (q, C4) 151.12 (CH, C2) 148.69 (q, C6) 126.77 (q, C5) 110.08 (q, C8) 92.43 (CH, C1’) 86.28 (CH, C4’) 70.68 (CH, C2’) 70.63 (CH, C3’) 61.89 (CH_2, C5’) 34.39 (CH, $CH(CH_3)_2$) 19.24 (CH_3, $CH(CH_3)_2$) 19.21 (CH_3, $CH(CH_3)_2$), MALDI-TOF *m/z* 463.93 ($[M + H]^+$, $C_{14}H_{18}IN_5O_5H^+$ calc. 464.04)

5´-*O*-(4,4´-Dimethoxytrityl)-6-*N*-isobutyryl-8-iodoadenosine (6)

Preparation of **6** was achieved analogous as described in [3].

6-*N*-Isobutyryl-8-iodoadenosine (**5**, 0.8 g, 1.8 mmol) was dissolved in dry pyridine (15 ml, freshly distilled), followed by addition of DMT-Cl (0.7 g, 2.2 mmol). The mixture was stirred at room temperature for 1.5 h, and the reaction was stopped by addition of MeOH (5 ml). The solvent was removed under reduced pressure, and the residue was coevaporated three times with toluene before resolving it in DCM. The solution was washed with saturated aq. $NaHCO_3$ twice and the combined aq. phase was extracted with ‘DCM. The combined organic phase was dried with Na_2SO_4 and evaporated to dryness. After column chromatography (DCM/MeOH 99:1 to 97:3) compound **6** was obtained as a white solid (1.1 g, 1.5 mmol, 83%).

R_f 0.45 (DCM/MeOH 96:4), ^{1}H-NMR (600 MHz, DMSO-d_6): δ 10.73 (1H, s, NH) 8.41 (1H, s, H2) 7.27 (2H, d, *J* 7.7 Hz, DMT) 7.17 (7H, m, DMT) 6.79 (2H, d, *J* 9 Hz, DMT) 6.75 (2H, d, *J* 9 Hz, DMT) 5.86 (1H, d, *J* 4.5 Hz, H1‘) 5.54 (1H, d, *J* 5.7 Hz, 2‘OH) 5.32 (1H, dd, *J* 5.1 Hz, H2‘) 5.28 (1H, d, *J* 5.9 Hz, 3‘OH) 4.55 (1H, dd, *J* 5.5 Hz, H3’) 4.08 (1H, dd, *J* 4.8 Hz, H4’) 3.71 (3H, s, OCH_3) 3.70 (3H, s, OCH_3) 3.24 (1H, m, H5‘(a)) 3.12 (1H, m, H5‘(b)) 2.94 (1H, sept, *J* 6.8 Hz, C**H**$(CH_3)_2$) 1.12 (6H, d, *J* 6.8 Hz, $CH(CH_3)_2$) ^{13}C-NMR (150 MHz, DMSO-d_6): δ 175.24 (q, C=O) 157.96 (q, DMT) 157.92 (q, DMT) 152.02 (q, C4) 151.14 (CH, C2) 148.51 (q, C6) 144.91 (q, DMT) 135.64 (q, DMT) 135.56 (q, DMT) 129.66 (CH, DMT) 129.55 (CH, DMT) 127.65 (CH, DMT) 127.59 (CH, DMT) 126.51 (CH, DMT 126.38 (q, C5) 113.03 (CH, DMT) 112.98 (CH, DMT) 110.27 (q, C8) 92.73 (CH, C1’) 85.19 (q, DMT) 83.25 (CH, C4’) 70.65 (CH, C2’) 70.17 (CH, C3’) 63.14 (CH_2, C5’) 54.97 (OCH_3) 54.96 (OCH_3) 34.39 (CH, $CH(CH_3)_2$) 19.24 (CH_3, $CH(CH_3)_2$) 19.21 (CH_3, $CH(CH_3)_2$), MALDI-TOF *m/z* 765.89 ($[M + H]^+$, $C_{35}H_{36}IN_5O_7H^+$ calc. 766.17)

5´-*O*-(4,4´-Dimethoxytrityl)-6-*N*-Isobutyryl-8-(3-[6-trifluoroacetamidohexanamide]prop-2-ynyl)-adenosine (7)

5´-*O*-(4,4´-Dimethoxytrityl)-6-*N*-isobutyryl-8-iodoadenosine (**6**, 0.8 g, 1.1 mmol) was filled into a Schlenk flask together with $Pd(PPh_3)_4$ (0.12 g, 0.11 mmol), CuI (0.04 g, 0.22 mmol) and *N*-(propyn-2-yl)-6-(trifluoroacetamido)hexanamide (**L**, 0.34 g, 1.3 mmol), purged with argon three times, and dissolved in 10 ml dry DMF. Afterwards, TEA (450 µl, 3.2 mmol, freshly

distilled) was added dropwise to the clear yellow solution to start the reaction. The reaction mixture was stirred at room temperature for 19 h. Reaction was stopped by addition of 5 ml ethyl acetate, and the solvents were removed under reduced pressure. The residue was resolved in DCM and the resulting solution was washed with saturated aq. $NaHCO_3$ twice. The combined aq. phase was extracted with ‘DCM. The combined organic phase was dried with Na_2SO_4 and evaporated to dryness. After column chromatography (DCM/MeOH 99:1 to 97:3) compound **7** was obtained as a white solid (0.51 g, 0.6 mmol, 53%).

R_f 0.35 (DCM/MeOH 96:4), ^{1}H-NMR (600 MHz, DMSO-d$_6$): *δ* 10.77 (1H, s, NH) 9.40 (1H, t, *J* 5.3 Hz, NH) 8.52 (1H, t, *J* 5.5 Hz, NH) 8.48 (1H, s, H2) 7.30 (2H, d, *J* 8.1 Hz, DMT) 7.17 (7H, m, DMT) 6.79 (2H, d, *J* 8.9 Hz, DMT) 6.75 (2H, d, *J* 8.9 Hz, DMT) 6.02 (1H, d, *J* 4.8 Hz, H1‘) 5.52 (1H, d, *J* 5.6 Hz, 2‘OH) 5.27 (1H, d, *J* 5.7 Hz, 3‘OH) 5.15 (1H, dd, *J* 5.1 and 10.2 Hz, H2‘) 4.51 (1H, dd, *J* 5.3 and 10.6 Hz, H3’) 4.24 (2H, d, *J* 5.5 Hz, NH-CH$_2$-C≡C) 4.09 (1H, dd, *J* 4.9 and 9.7 Hz, H4’) 3.71 (3H, s, OCH_3) 3.69 (3H, s, OCH_3) 3.21 (1H, m, H5‘(a)) 3.17 (3H, m, H5‘(b)/NH-**CH$_2$**-CH$_2$) 2.94 (1H, sept, *J* 6.8 Hz, C**H**(CH_3)$_2$) 2.15 (2H, t, *J* 7.6 Hz, CH$_2$-C=O) 1.53 (2H, m, CH$_2$) 1.48 (2H, m, CH$_2$) 1.25 (2H, m, CH$_2$) 1.12 (6H, d, *J* 6.8 Hz, CH(CH_3)$_2$) ^{13}C-NMR (150 MHz, DMSO-d$_6$): *δ* 175.23 (q, C=O) 172.14 (q, C=O) 157.97 (q, DMT) 157.93 (q, DMT) (156.47 156.24 155.99 155.76, q, *J* 37 Hz, (**C**=O)CF_3) 152.36 (CH, C2) 150.81 (q, C4) 149.87 (q, C6) 144.93 (q, DMT) 136.55 (q, C8) 135.61 (q, DMT) 135.58 (q, DMT) 129.68 (CH, DMT) 129.59 (CH, DMT) 127.64 (CH, DMT) 127.61 (CH, DMT) 126.51 (CH, DMT) 123.60 (q, C5) (118.85 117.00 115.05 113.12, q, *J* 288 Hz, (C=O)**C**F$_3$) 113.01 (CH, DMT) 112.96 (CH, DMT) 95.49 (q, C≡C) 89.77 (CH, C1’) 85.24 (q, DMT) 83.56 (CH, C4’) 71.13 (CH, C2’) 70.42 (q, C≡C) 70.28 (CH, C3’) 63.48 (CH$_2$, C5’) 54.96 (OCH_3) 54.94 (OCH_3) 39.03 (CH$_2$,) 34.91 (CH$_2$)34.43 (CH, CH(CH_3)$_2$) 28.51 (CH$_2$, NH-CH$_2$-C≡C) 28.01 (CH$_2$) 25.85 (CH$_2$) 24.64 (CH$_2$) 19.20 (CH_3, CH(CH_3)$_2$) 19.18 (CH_3, CH(CH_3)$_2$), MALDI-TOF *m/z* 901.74 ([M + H]$^+$, $C_{46}H_{50}F_3N_7O_9H^+$ calc. 902.36)

3',5'-*O*-(Di-*tert*-butylsilylene)-2'-*O*-(*tert*-butyldimethylsilyl)-adenosine (10)

Synthesis of compound **10** was described before [4].

Adenosine (0.5 g, 1.9 mmol) was dried by evaporation with dry DCM three times, purged with argon and dissolved in 4 ml dry DMF. The reaction mixture was cooled down to 0 °C, followed by the dropwise addition of di-*tert*-butylsilandiyltriflate (0.73 ml, 2.2 mmol), and the reaction mixture was stirred for 45 min at 0 °C. Afterwards, imidazole (0.64 g, 9.3 mmol) and TBDMS-Cl (0.42 g, 2.8 mmol) was added and the reaction mixture was stirred for 12 h at room temperature. DMF was removed under reduced pressure, the residue was resolved in DCM and the resulting solution was washed with saturated aq. $NaHCO_3$ twice. The combined aq. phase was extracted with DCM. The combined organic phase was dried with Na_2SO_4 and evaporated to dryness. After column chromatography (DCM/MeOH 99:1 to 95:5) compound **10** was obtained as a colourless solid (0.8 g, 1.6 mmol, 83%).

R_f 0.70 (‘DCM/MeOH 95:5), ^{1}H-NMR (600 MHz, DMSO-d$_6$): *δ* 8.34, 8.13 (2H, s, s, H2/H8) 7.35 (2H, br s, NH$_2$) 5.95 (1H, s, H1‘) 4.73 (1H, m, H2’) 4.67 (1H, d, *J* 5.1 Hz, H3’) 4.36 (1H, d, *J* 4.5 Hz, H4‘) 3.99 (2H, d, *J* 6.8 Hz, H5‘(a)/H5‘(b)) 1.08 (9H, s, SiC(CH_3)$_3$) 1.01 (9H, s, SiC(CH_3)$_3$) 0.86

(9H, s, $SiC(CH_3)_3$) 0.09 (3H, s, $SiCH_3$) 0.07 (3H, s, $SiCH_3$) ^{13}C-NMR (150 MHz, DMSO-d_6): δ 156.08 (q, C4) 152.68 (CH, C2) 148.77 (q, C6) 139.78 (CH, C8) 119.07 (q, C5) 91.00 (CH, C1’) 75.21 (CH, C4’) 74.81 (CH, C2’) 74.08 (CH, C3’) 67.02 (CH_2, C5’) 27.33 (CH_3, $SiC(CH_3)_3$) 26.83 (CH_3, $SiC(CH_3)_3$) 25.69 (CH_3, $SiC(CH_3)_3$) 22.22 (q, $SiC(CH_3)_3$) 19.97 (q, $SiC(CH_3)_3$) 18.07 (q, $SiC(CH_3)_3$) -4.58 ($SiCH_3$) -5.18 ($SiCH_3$), MALDI-TOF *m/z* 522.08 ([M + H]$^+$, $C_{24}H_{43}N_5O_4Si_2H^+$ calc. 522.29)

3',5'-*O*-(Di-*tert*-butylsilylene)-2'-*O*-(*tert*-butyldimethylsilyl)-8-iodoadenosine (11)

As described for **3**, with **10** (5.6 g, 10.7 mmol), 2 M LDA (27 ml, 54 mmol), iodine (4.9 g, 19.3 mmol) and acetic acid (3 ml, 54 mmol). Purification was done via column chromatography (hexane/ ethyl acetate 3:1 to 1:1), and compound **11** was obtained as a white solid (5.8 g, 8.9 mmol, 83%).

R_f 0.50 (hexane/ethyl acetate 1:1), ^{1}H-NMR (600 MHz, DMSO-d_6): δ 8.03 (1H, s, H2) 7.48 (2H, br s, NH_2) 5.74 (1H, s, H1‘) 5.18 (1H, dd, *J* 5.4 Hz, H2’) 4.90 (1H, d, *J* 5.4 Hz, H3’) 4.34 (1H, dd, *J* 5 Hz, H4‘) 3.95, 3.87 (2H, m/m, H5‘(a)/H5‘(b)) 1.08 (9H, s, $SiC(CH_3)_3$) 1.01 (9H, s, $SiC(CH_3)_3$) 0.86 (9H, s, $SiC(CH_3)_3$) 0.073 (3H, s, $SiCH_3$) 0.071 (3H, s, $SiCH_3$) ^{13}C-NMR (150 MHz, DMSO-d_6): δ 154.91 (q, C4) 152.66 (CH, C2) 149.70 (q, C6) 121.95 (q, C5) 103.53 (q, C8) 95.08 (CH, C1’) 74.61 (CH, C4’) 74.25 (CH, C2’) 73.62 (CH, C3’) 66.92 (CH_2, C5’) 27.28 (CH_3, $SiC(CH_3)_3$) 26.80 (CH_3, $SiC(CH_3)_3$) 25.66 (CH_3, $SiC(CH_3)_3$) 22.27 (q, $SiC(CH_3)_3$) 19.93 (q, $SiC(CH_3)_3$) 18.13 (q, $SiC(CH_3)_3$) -4.49 ($SiCH_3$) -5.30 ($SiCH_3$), MALDI-TOF *m/z* 648.01 ([M + H]$^+$, $C_{24}H_{42}IN_5O_4Si_2H^+$ calc. 648.18)

3',5'-*O*-(Di-*tert*-butylsilylene)-2'-*O*-(*tert*-butyldimethylsilyl)-6-*N*-isobutyryl-8-iodoadenosine (12)

As described for **4**, with **11** (1.6 g, 2.5 mmol) and isobuytric anhydride (2.5 ml, 15 mmol). Purification was done via column chromatography (hexane/ ethyl acetate 9:1 to 8:2), and compound **12** was obtained as a white solid (1 g, 1.4 mmol, 57%).

R_f 0.45 (hexane/ethyl acetate 8:2), ^{1}H-NMR (600 MHz, DMSO-d_6): δ 10.78 (1H, s, NH) 8.54 (1H, s, H2) 5.84 (1H, s, H1‘) 5.19 (1H, dd, *J* 5.4 Hz, H2’) 4.94 (1H, d, *J* 5.3 Hz, H3’) 4.37 (1H, dd, *J* 5.2 Hz, H4‘) 4.01, 3.92 (2H, m/m, H5‘(a)/H5‘(b)) 2.93 (1H, sept, *J* 6.8 Hz, C**H**$(CH_3)_2$) 1.11 (6H, d, *J* 6.8 Hz, $CH(CH_3)_2$) 1.09 (9H, s, $SiC(CH_3)_3$) 1.02 (9H, s, $SiC(CH_3)_3$) 0.87 (9H, s, $SiC(CH_3)_3$) 0.08 (6H, s, $SiCH_3$) ^{13}C-NMR (150 MHz, DMSO-d_6): δ 175.20 (q, C=O) 151.82 (q, C4) 151.56 (CH, C2) 148.72 (q, C6) 126.46 (q, C5) 109.38 (q, C8) 95.34 (CH, C1’) 74.65 (CH, C4’) 74.39 (CH, C2’) 73.67 (CH, C3’) 66.88 (CH_2, C5’) 34.39 (CH, $CH(CH_3)_2$) 27.31 (CH_3, $SiC(CH_3)_3$) 26.81 (CH_3, $SiC(CH_3)_3$) 25.69 (CH_3, $SiC(CH_3)_3$) 22.30 (q, $SiC(CH_3)_3$) 19.94 (q, $SiC(CH_3)_3$) 19.19 (CH_3, $CH(CH_3)_2$) 18.09 (q, $SiC(CH_3)_3$) -4.47 ($SiCH_3$) -5.26 ($SiCH_3$), MALDI-TOF *m/z* 717.96 ([M + H]$^+$, $C_{28}H_{48}IN_5O_5Si_2H^+$ calc. 718.22)

5´-*O*-(4,4´-Dimethoxytrityl)-2'-*O*-(*tert*-butyldimethylsilyl)-6-*N*-Isobutyryl-8-iodoadenosine (13)

3',5'-*O*-(Di-*tert*-butylsilylene)-2'-*O*-(*tert*-butyldimethylsilyl)-6-*N*-isobutyryl-8-iodoadenosine **12** (1 g, 1.4 mmol) was dissolved in 6 ml dry DCM, followed by the addition of pyridine (1 ml,

fresh distilled) and cooling of the solution to 0 °C. The addition of the 70% HF pyridine solution (100 µl, 5.5 mmol) occurred dropwise, and the resulting reaction mixture was stirred at 0 °C for 3 h. Reaction was stopped by addition of 10 ml saturated $NaHCO_3$ solution. The addition of 30 ml ethyl acetate took place after 5 min, the organic phase was washed three times with a saturated $NaHCO_3$ solution, dried with Na_2SO_4 and evaporated to dryness. The intermediate product was coevaporated three times with dry pyridine and dissolved in 11.5 ml dry pyridine, followed by addition of DMT-Cl (0.6 g, 1.8 mmol). The mixture was stirred at room temperature for 1.5 h, and the reaction was stopped by addition of MeOH (5 ml). The solvent was removed under reduced pressure, and the residue was coevaporated three times with toluene before resolving it in DCM. The solution was washed with saturated aq. $NaHCO_3$ twice and the combined aq. phase was extracted with 'DCM. The combined organic phase was dried with Na_2SO_4 and evaporated to dryness. After column chromatography (hexane/ethyl acetate 3:1 to 2:1) compound **13** was obtained as a white solid (0.75 g, 0.86 mmol, 62% over two steps).

R_f 0.36 (hexane/ethyl acetate 2:1), ^{1}H-NMR (600 MHz, DMSO-d$_6$): δ 10.76 (1H, s, NH) 8.37 (1H, s, H2) 7.35 (2H, d, *J* 8.2 Hz, DMT) 7.19 (7H, m, DMT) 6.82 (2H, d, *J* 9 Hz, DMT) 6.79 (2H, d, *J* 9 Hz, DMT) 5.88 (1H, d, *J* 5.2 Hz, H1') 5.36 (1H, dd, *J* 5.2 Hz, H2') 5.24 (1H, d, *J* 6.1 Hz, 3'OH) 4.44 (1H, dd, *J* 5.4 Hz, H3') 4.12 (1H, dd, *J* 4.6 Hz, H4') 3.71 (3H, s, -OCH_3) 3.70 (3H, s, -OCH_3) 3.31, 3.18 (2H, m/m, H5'(a)/H5'(b)) 2.93 (1H, sept, *J* 6.8 Hz, C**H**$(CH_3)_2$) 1.12 (6H, d, *J* 6.8 Hz, $CH(CH_3)_2$) 0.74 (9H, s, $SiC(CH_3)_3$) -0.09 (3H, s, $SiCH_3$) -0.22 (3H, s, $SiCH_3$) ^{13}C-NMR (150 MHz, DMSO-d$_6$): δ 175.21 (q, C=O) 157.99 (q, DMT) 157.97 (q, DMT) 151.99 (q, C4) 151.14 (CH, C2) 148.59 (q, C6) 144.95 (q, DMT) 135.56 (q, DMT) 129.71 (CH, DMT) 129.64 (CH, DMT) 127.68 (CH, DMT) 127.62 (CH, DMT) 126.55 (CH, DMT) 126.39 (q, C5) 113.04 (CH, DMT) 113.03 (CH, DMT) 110.02 (q, C8) 92.49 (CH, C1') 85.28 (q, DMT) 83.77 (CH, C4') 72.30 (CH, C2') 70.07 (CH, C3') 62.88 (CH_2, C5') 54,99 (CH, OCH_3) 54,97 (CH, OCH_3) 34,41 (CH, $CH(CH_3)_2$) 25.55 (CH_3, $SiC(CH_3)_3$) 19.24 (CH_3, $CH(CH_3)_2$) 19.19 (CH_3, $CH(CH_3)_2$) 17.81 (q, $SiC(CH_3)_3$) -4.47 ($SiCH_3$) -5.26 ($SiCH_3$), MALDI-TOF *m/z* 880.28 ([M + H]$^+$, $C_{41}H_{50}IN_5O_7H^+$ calc. 880.25)

5´-*O*-(4,4´-Dimethoxytrityl)-2'-*O*-(*tert*-butyldimethylsilyl)-6-*N*-isobutyryl-8-(3-[6-trifluoroacetamidohexanamide]prop-2-ynyl)-adenosine (8)

As described for **7** with nucleoside derivate **13** (0.73 g, 0.83 mmol), $Pd(PPh_3)_4$ (0.1 g, 0.08 mmol), CuI (0.03 g, 0.16 mmol) and *N*-(propyn-2-yl)-6-(trifluoroacetamido)hexanamide (0.26 g, 1 mmol) in 8 ml dry DMF and TEA (345 µl, 3 mmol, fresh distilled). The residue was resolved in ethyl acetate, the resulting solution was washed with saturated aq. $NaHCO_3$ twice and the combined aq. phase was extracted with DCM. The combined organic phase was dried with Na_2SO_4 and evaporated to dryness. After column chromatography (hexane/ethyl acetate 1:1 to 1:3) compound **8** was obtained as a white solid (0.43 g, 0.42 mmol, 51%).

R_f 0.35 (hexane/ethyl acetate 1:3), ^{1}H-NMR (600 MHz, DMSO-d$_6$): δ 10.79 (1H, s, NH) 9.40 (1H, t, J 5.6 Hz, NH) 8.49 (2H, m, NH/H2) 7.36 (2H, d, *J* 8 Hz, DMT) 7.21 (6H, m, DMT) 7.17 (1H, m, DMT) 6.81 (2H, d, *J* 8.9 Hz, DMT) 6.78 (2H, d, *J* 8.9 Hz, DMT) 6.03 (1H, d, *J* 5.0 Hz, H1') 5.17 (2H,

m, H2’/3’OH) 4.46 (1H, dd, *J* 5.3 Hz, H3’) 4.19 (2H, d, *J* 5.3 Hz, NH-CH_2-C≡C) 4.12 (1H, dd, *J* 4.8 Hz, H4‘) 3.71 (3H, s, -OCH_3) 3.70 (3H, s, -OCH_3) 3.29, 3.23 (2H, m/m, H5‘(a)/H5‘(b)) 3.16 (2H, dd, *J* 6.8 Hz, NH-**CH_2**-CH_2) 2.93 (1H, sept, *J* 6.8 Hz, $CH(CH_3)_2$) 2.13 (2H, t, *J* 7.6 Hz, CH_2-C=O) 1.52 (2H, m, *J* 7.6 Hz, CH_2) 1.48 (2H, quint, *J* 7.5 Hz, CH_2) 1.25 (2H, quint, *J* 7.5 Hz, CH_2) 1.12 (6H, d, *J* 6.8 Hz $CH(CH_3)_2$) 0.73 (9H, s, $SiC(CH_3)_3$) -0.09 (3H, s, $SiCH_3$) -0.21 (3H, s, $SiCH_3$) ^{13}C-NMR (150 MHz, DMSO-d_6): δ 175.21 (q, C=O) 172.07 (q, C=O) 157.99 (q, DMT) 157.97 (q, DMT) (156.23 155.99, d, *J* 37 Hz, (**C**=O)CF_3) 152.41 (CH, C2) 150.81 (q, C4) 149.93 (q, C6) 144.95 (q, DMT) 136.23 (q, C8) 135.56 (q, DMT) 135.55 (q, DMT) 129.71 (CH, DMT) 129.66 (CH, DMT) 127.67 (CH, DMT) 127.64 (CH, DMT) 126.55 (CH, DMT) 123.55 (q, C5) 113.03 (CH, DMT) 113.00 (CH, DMT) 95.71 (q, C≡C) 89.53 (CH, C1’) 85.29 (q, DMT) 83.79 (CH, C4’) 72.99 (CH, C2’) 70.16 (q, C≡C) 70.09 (CH, C3’) 63.19 (CH_2, C5’) 54.97 (CH, OCH_3) 54.96 (CH, OCH_3) 39.02 (CH_2) 34.91 (CH_2) 34.44 (CH, $CH(CH_3)_2$) 28.45 (CH_2, NH-CH_2-C≡C) 28.01 (CH_2) 25.85 (CH_2) 25.50 (CH, $SiC(CH_3)_3$) 24.63 (CH_2) 19.20 (CH_3, $CH(CH_3)_2$) 19.16 (CH_3, $CH(CH_3)_2$) 17.79 (q, $SiC(CH_3)_3$) -4.84 ($SiCH_3$) -5.39 ($SiCH_3$), MALDI-TOF *m/z* 1016.86 ($[M + H]^+$, $C_{52}H_{64}F_3N_7O_9SiH^+$ calc. 1016.45)

5´-*O*-(4,4´-Dimethoxytrityl)-2'-*O*-(*tert*-butyldimethylsilyl)-6-*N*-isobutyryl-8-(3-[6-trifluoroacetamidohexanamide]prop-2-ynyl)adenosine-3’-O-(cyanoethyl-N,N-diisopropylphosphoramidite) (9)

Preparation of **9** was achieved analogous as described in [3].

5´-*O*-(4,4´-Dimethoxytrityl)-2'-*O*-(*tert*-butyldimethylsilyl)-6-*N*-Isobutyryl-8-(3-[6-trifluoroacetylaminohexanamido]prop-1-ynyl)-adenosine (**8**, 0.2 g, 0.2 mmol) was dissolved in dry DCM (2.8 ml), followed by dropwise addition of TEA (111 µl, fresh distilled) and 2-cyanoethyl-*N*,*N*-diisopropylchlorophosphoramidite (54 µl). After stirring at room temperature for 1 h, progress of the reaction was controlled by TLC, showing that no starting material was left. Reaction was stopped by addition of 20 ml DCM (stored over $NaHCO_3$), and the resulting solution was washed with saturated $NaHCO_3$. The organic phase was dried with Na_2SO_4 and evaporated to dryness. The residue was subjected to column chromatography (DCM/acetone/TEA 95:4:1) yielding the nucleoside phosphoramidite **9** as a colourless oil (0.13 g, 0.11 mmol, 52%). R_f 0.2 (DCM/acetone/TEA 95:4:1); ^{31}P-NMR (240 MHz, $CDCl_3$): δ 151.29 (s), 148.29 (s).

References

1. Rublack, N.; Nguyen, H.; Appel, B.; Springstubbe, D.; Strohbach, D.; Müller, S., *Journal of nucleic acids*, **2011**, Article ID 805253, 19 pages
2. Nair, V.; Chamberlain, S. D., *J Org Chem* **1985,** *50*, 5069-5075.
3. Frommer, J.; Karg, B.; Weisz, K.; Müller, S., *Org Biomol Chem* **2018,** *16* (41), 7663-7673.
4. Shishodia, S.; Zhang, D.; El-Sagheer, A. H.; Brown, T.; Claridge, T. D. W.; Schofield, C. J.; Hopkinson, R. J., *Org Biomol Chem* **2018,** *16* (21), 4021-4032.

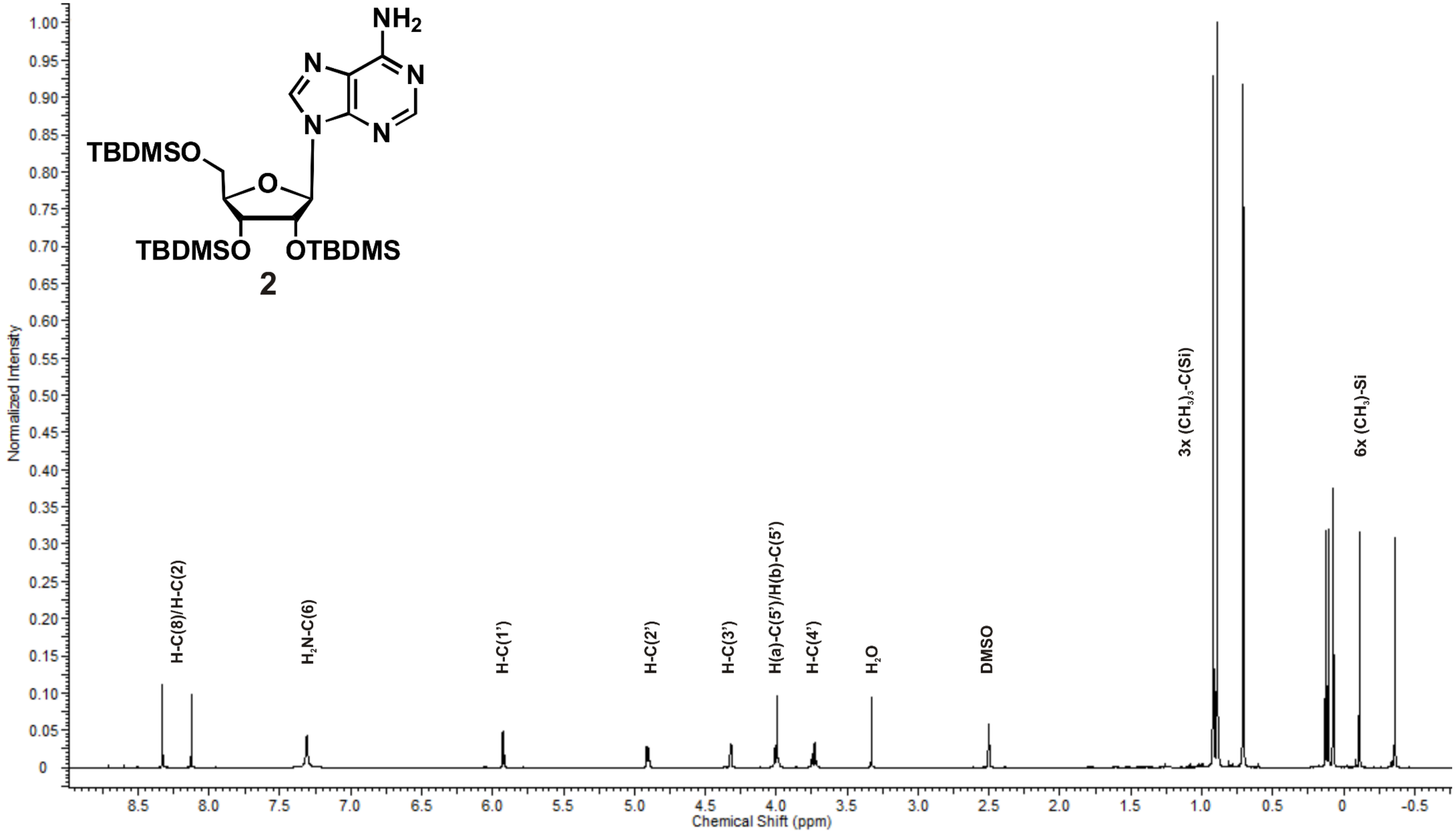
NH2
N
N
N
N
TBDMSO
O
TBDMSO
OTBDMS
2
Normalized Intensity
Chemical Shift (ppm)
H-C(8)/H-C(2)
H2N-C(6)
H-C(1')
H-C(2')
H-C(3')
H(a)-C(5')/H(b)-C(5')
H-C(4')
H2O
DMSO
3x (CH3)3-C(Si)
6x (CH3)-Si

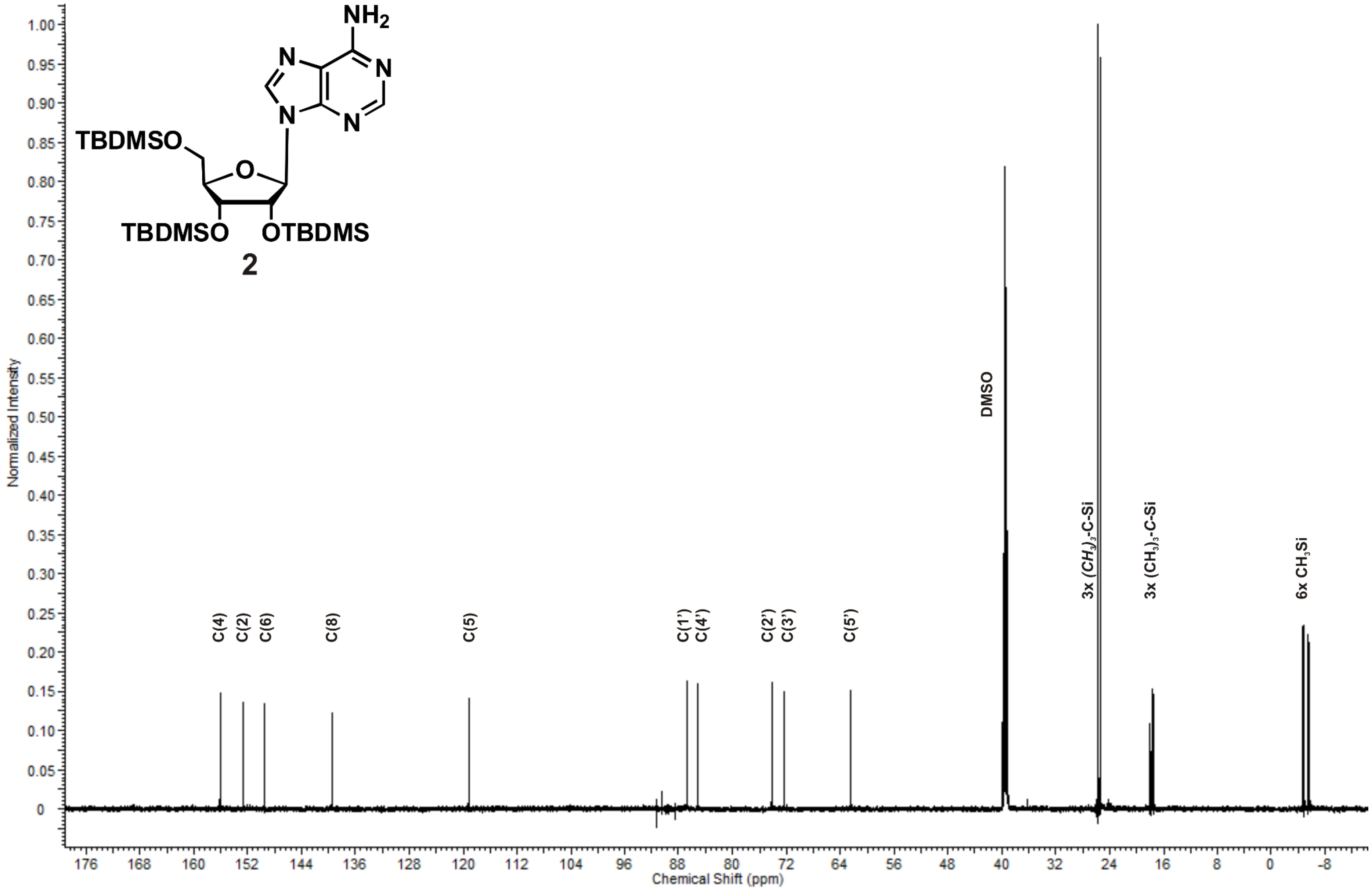
NH2
N
N
N
N
TBDMSO
O
TBDMSO
OTBDMS
2
DMSO
3x (CH3)3-C-Si
3x (CH3)3-C-Si
6x CH3Si
C(4)
C(2)
C(6)
C(8)
C(5)
C(1')
C(4')
C(2')
C(3')
C(5')
Normalized Intensity
Chemical Shift (ppm)

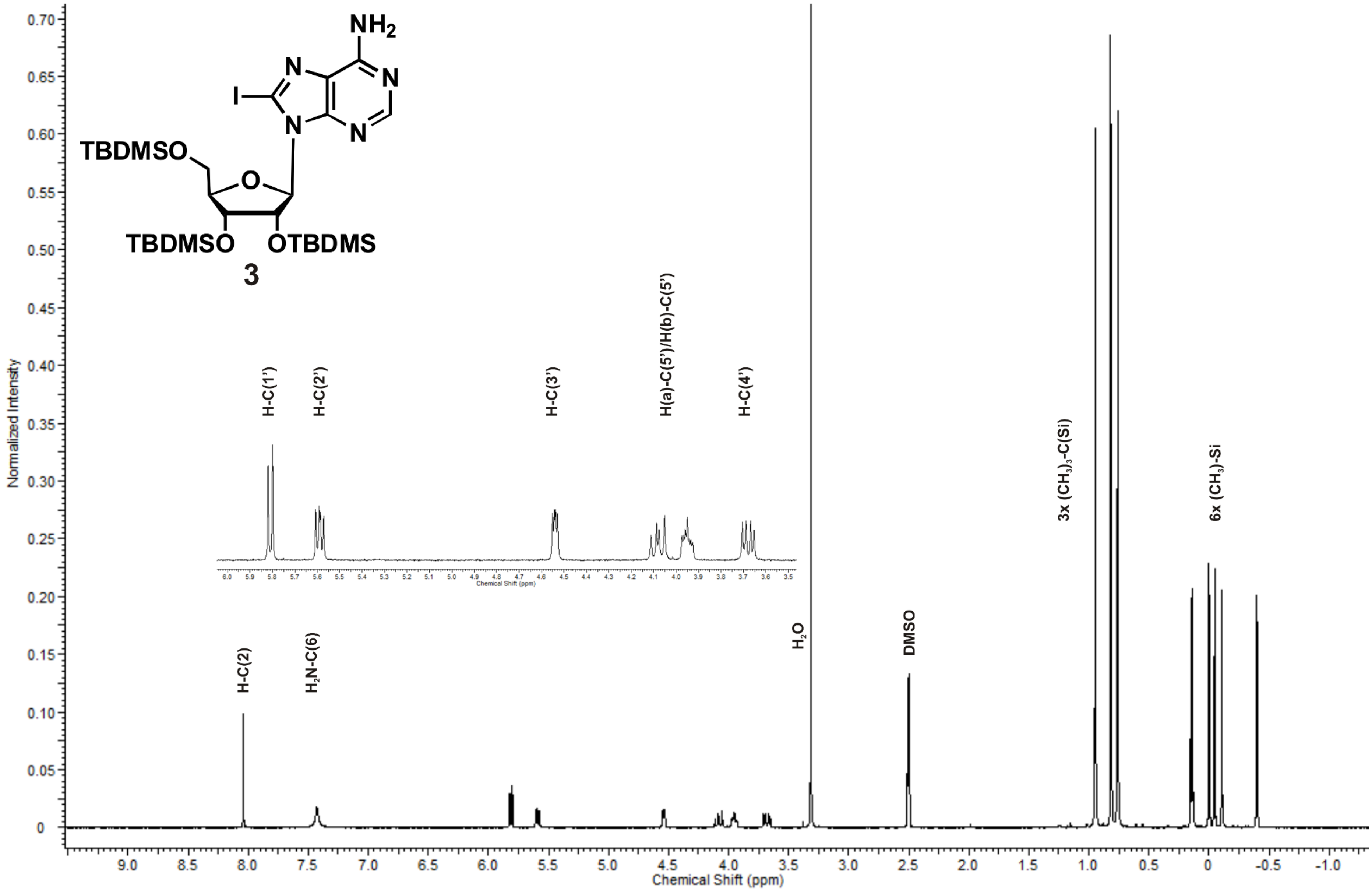
NH2
N
N
I
N
N
TBDMSO
O
TBDMSO
OTBDMS
3
H-C(1')
H-C(2')
H-C(3')
H(a)-C(5')/H(b)-C(5')
H-C(4')
Chemical Shift (ppm)
H-C(2)
H2N-C(6)
H2O
DMSO
3x (CH3)3-C(Si)
6x (CH3)-Si
Normalized Intensity
Chemical Shift (ppm)

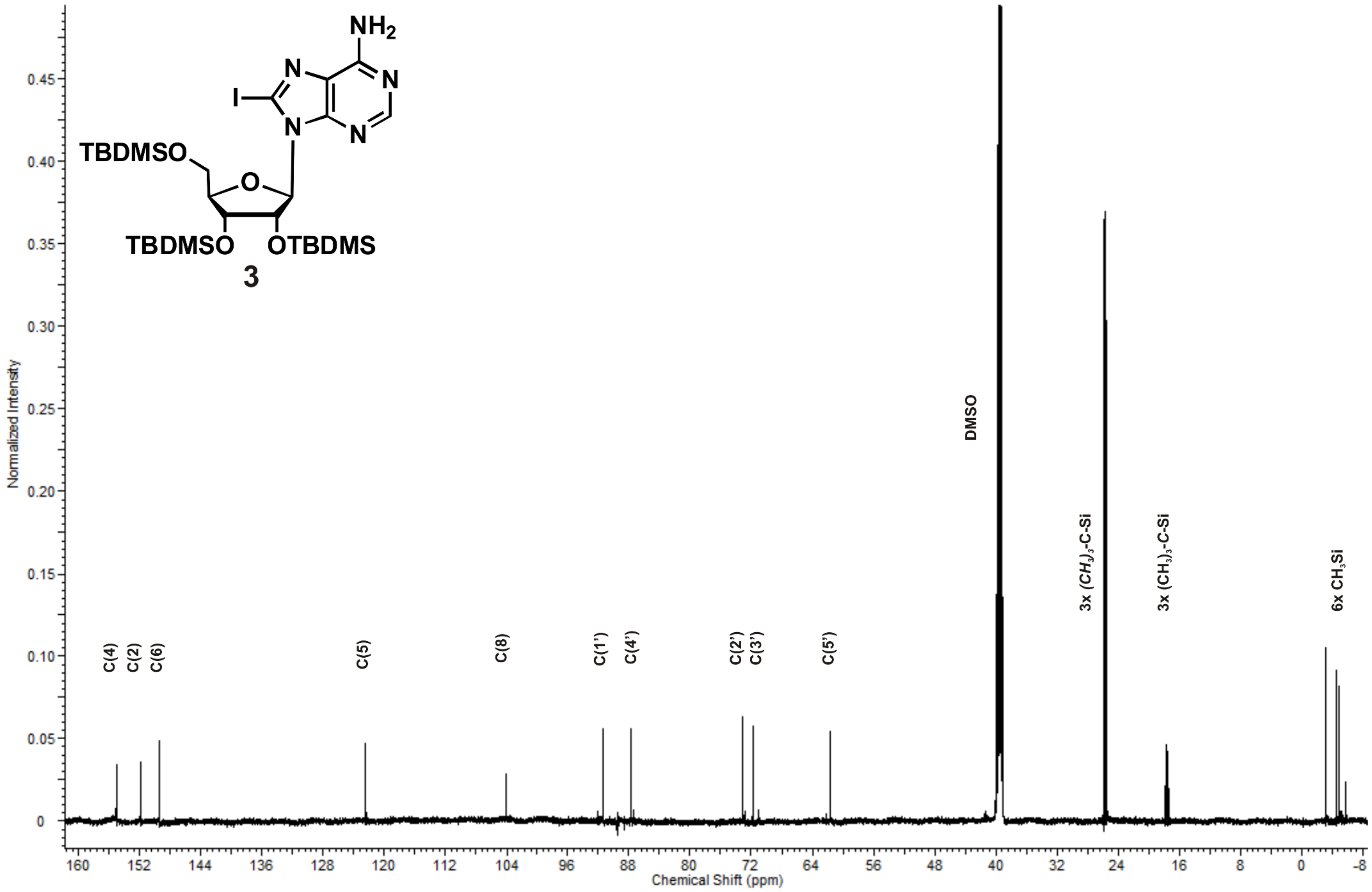

NH2
N
N
I
N
N
TBDMSO
O
TBDMSO
OTBDMS
3
Normalized Intensity
DMSO
3x (CH3)3-C-Si
3x (CH3)3-C-Si
6x CH3Si
C(4)
C(2)
C(6)
C(5)
C(8)
C(1')
C(4')
C(2')
C(3')
C(5')
Chemical Shift (ppm)

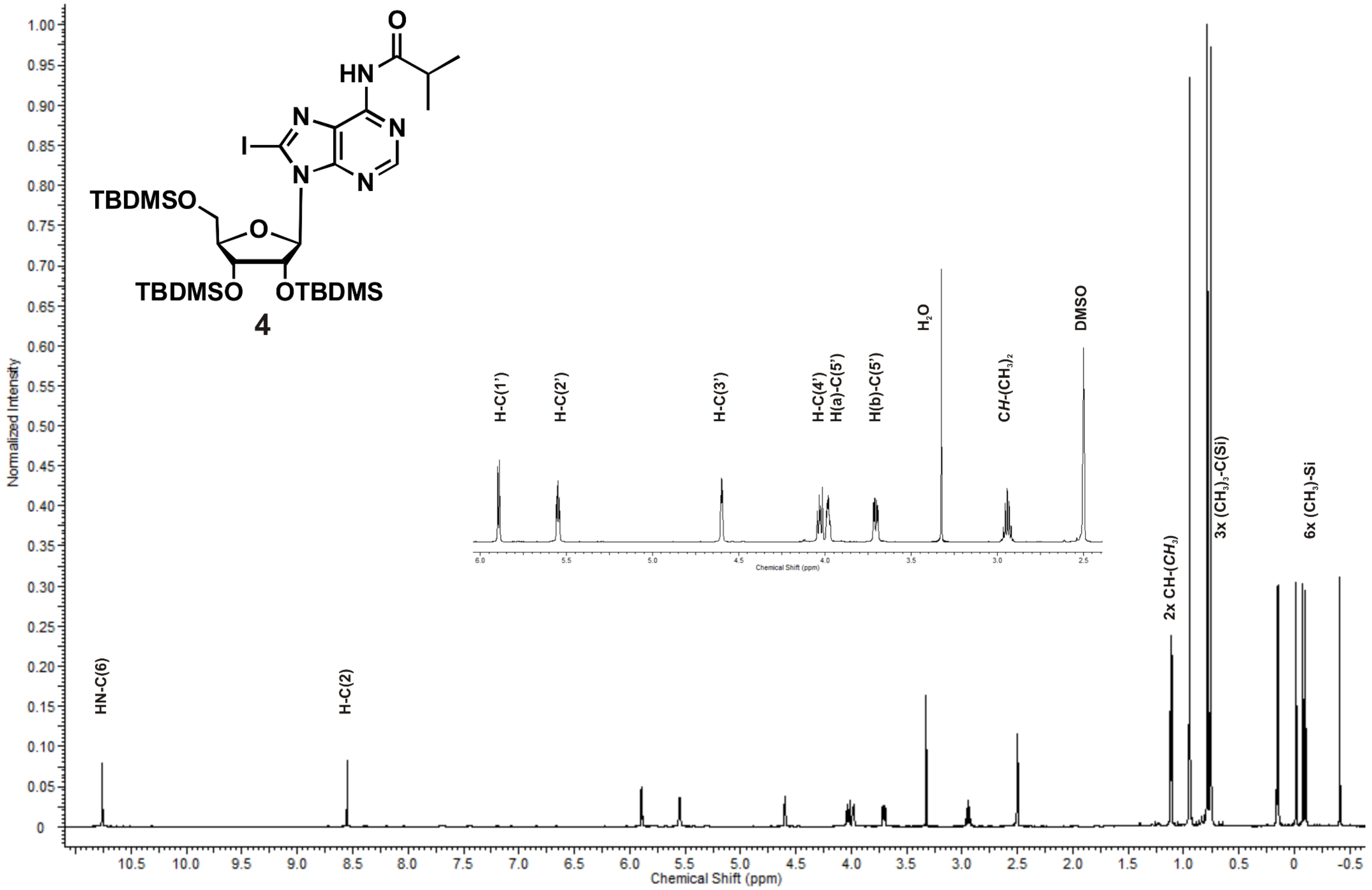

4
HN-C(6)
H-C(2)
H-C(1')
H-C(2')
H-C(3')
H-C(4')
H(a)-C(5')
H(b)-C(5')
H_2O
CH-$(CH_3)_2$
DMSO
2x CH-(CH_3)
3x $(CH_3)_3$-C(Si)
6x (CH_3)-Si
Normalized Intensity
Chemical Shift (ppm)

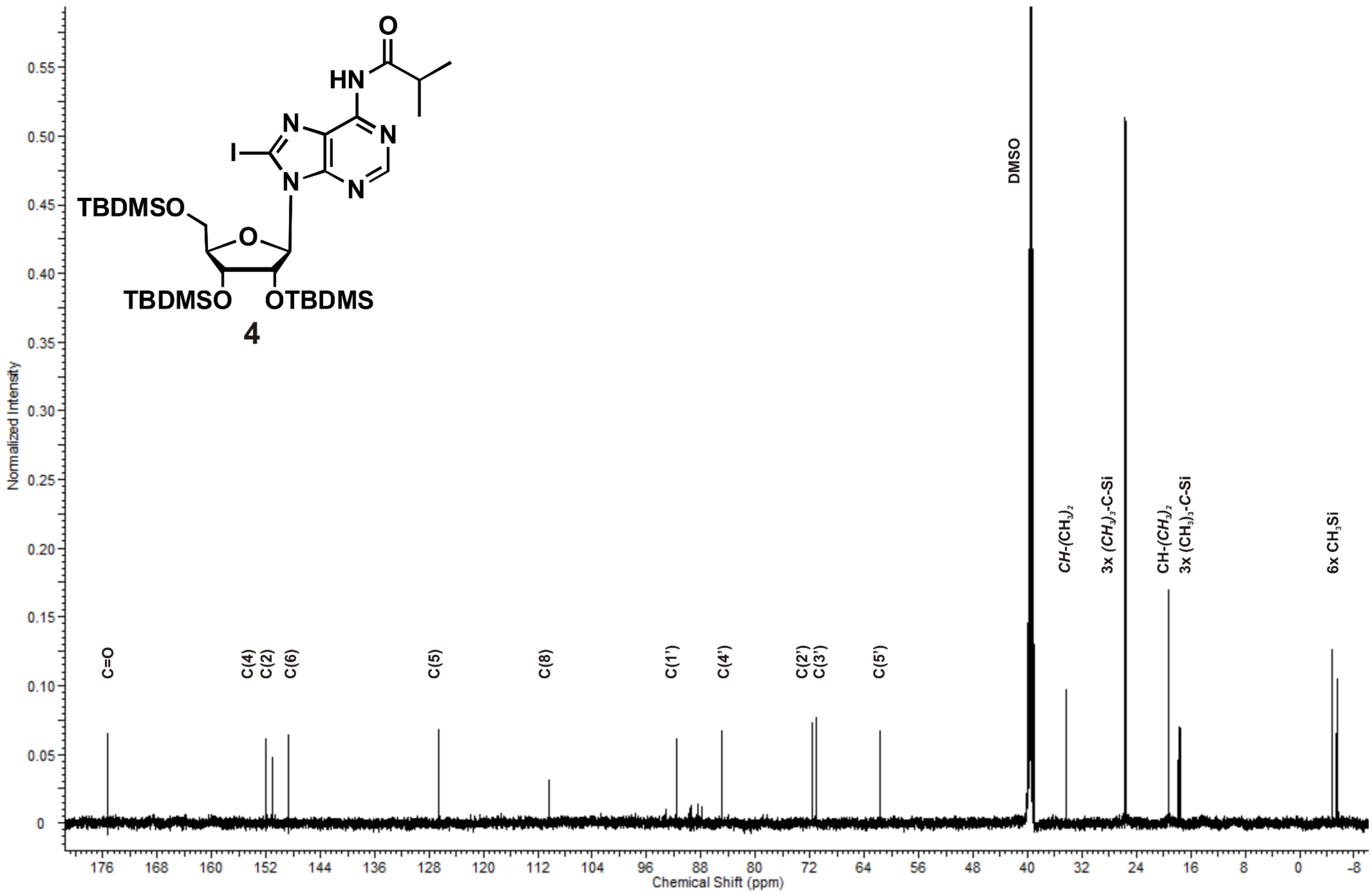

TBDMSO
TBDMSO
OTBDMS
HN
O
N
I
4
C=O
C(4)
C(2)
C(6)
C(5)
C(8)
C(1')
C(4')
C(2')
C(3')
C(5')
DMSO
CH-(CH3)2
3x (CH3)3-C-Si
CH-(CH3)2
3x (CH3)3-C-Si
6x CH3Si
Normalized Intensity
Chemical Shift (ppm)

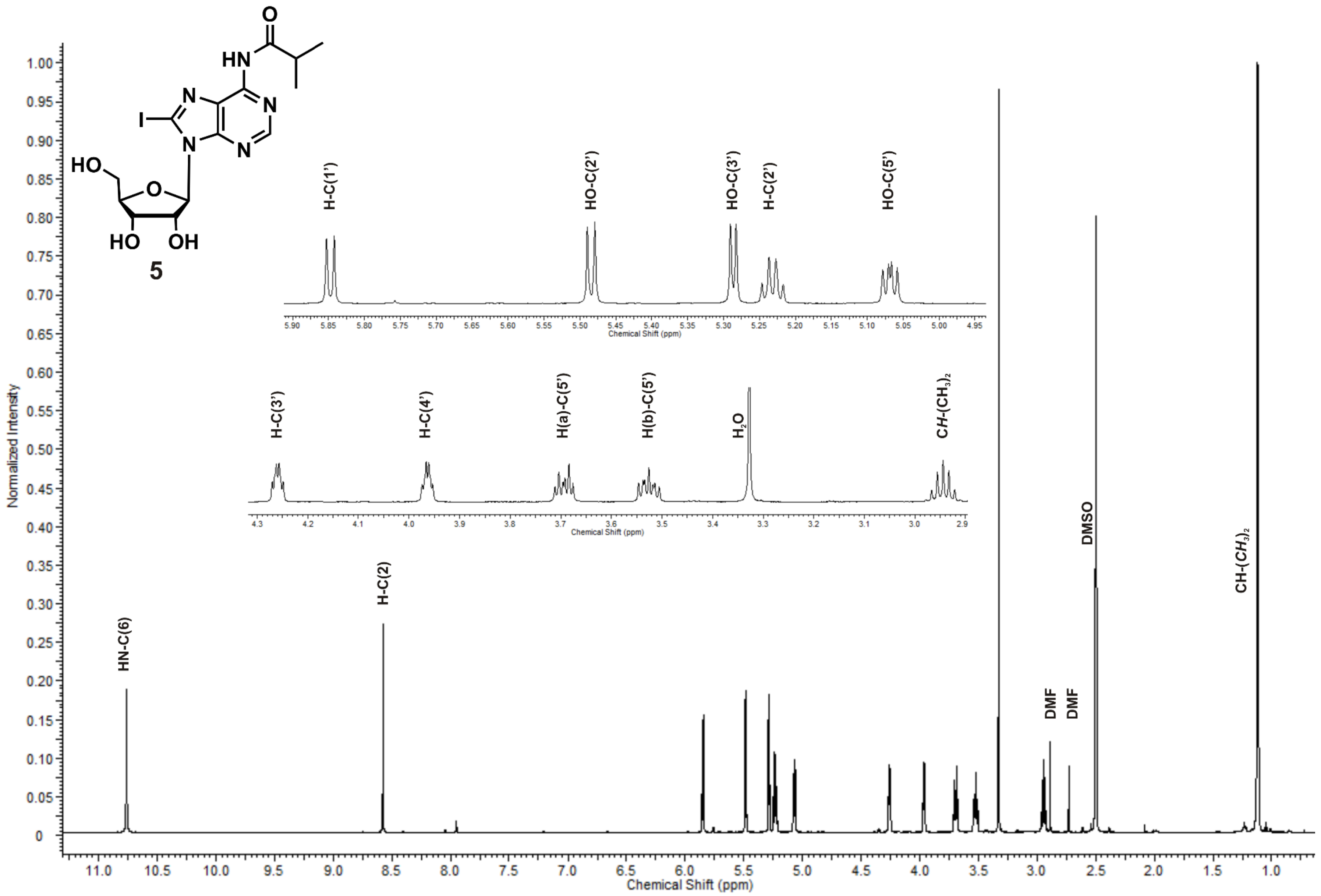
5
HN-C(6)
H-C(2)
H-C(1')
HO-C(2')
HO-C(3')
H-C(2')
HO-C(5')
H-C(3')
H-C(4')
H(a)-C(5')
H(b)-C(5')
H_2O
CH-(CH$_3$)$_2$
DMSO
DMF
DMF
CH-(CH$_3$)$_2$
Normalized Intensity
Chemical Shift (ppm)

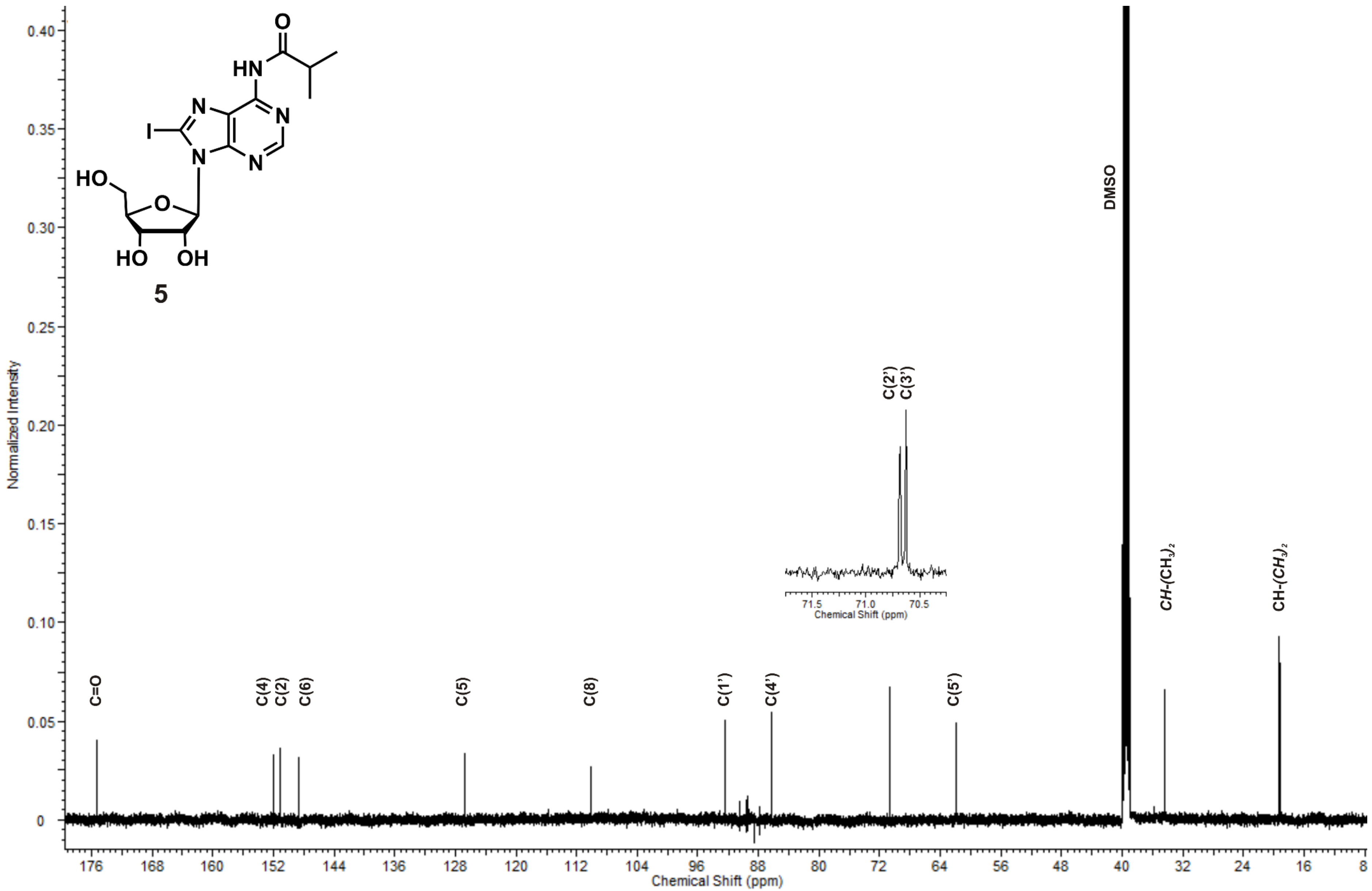

5
Normalized Intensity
DMSO
C(2')
C(3')
71.5
71.0
70.5
Chemical Shift (ppm)
CH-(CH3)2
CH-(CH3)2
C=O
C(4)
C(2)
C(6)
C(5)
C(8)
C(1')
C(4')
C(5')
Chemical Shift (ppm)

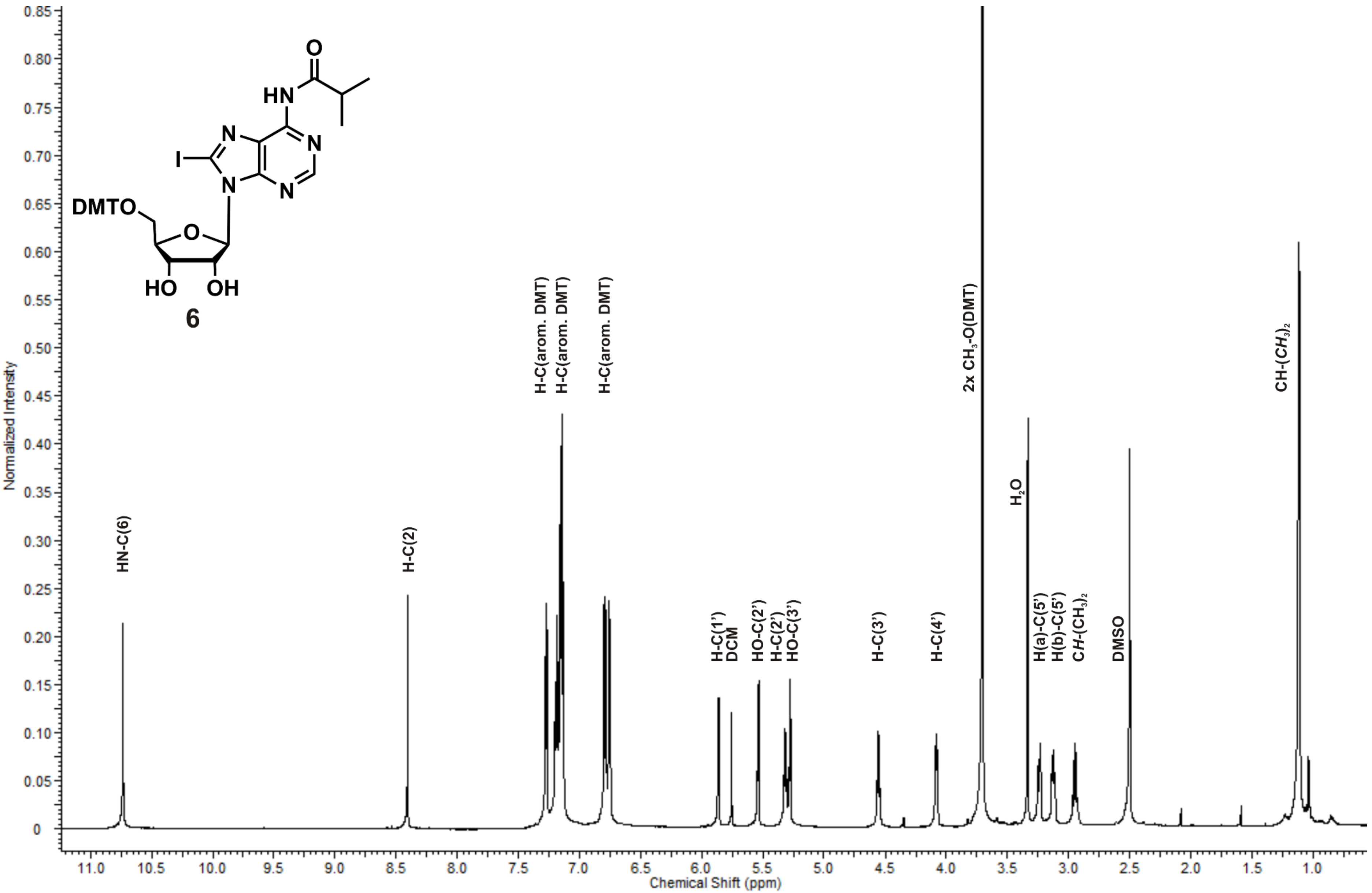

DMTO
HN
O
N
I
N
N
N
O
HO
OH
6
HN-C(6)
H-C(2)
H-C(arom. DMT)
H-C(arom. DMT)
H-C(arom. DMT)
H-C(1')
DCM
HO-C(2')
H-C(2')
HO-C(3')
H-C(3')
H-C(4')
2x CH3-O(DMT)
H2O
H(a)-C(5')
H(b)-C(5')
CH-(CH3)2
DMSO
CH-(CH3)2
Normalized Intensity
Chemical Shift (ppm)

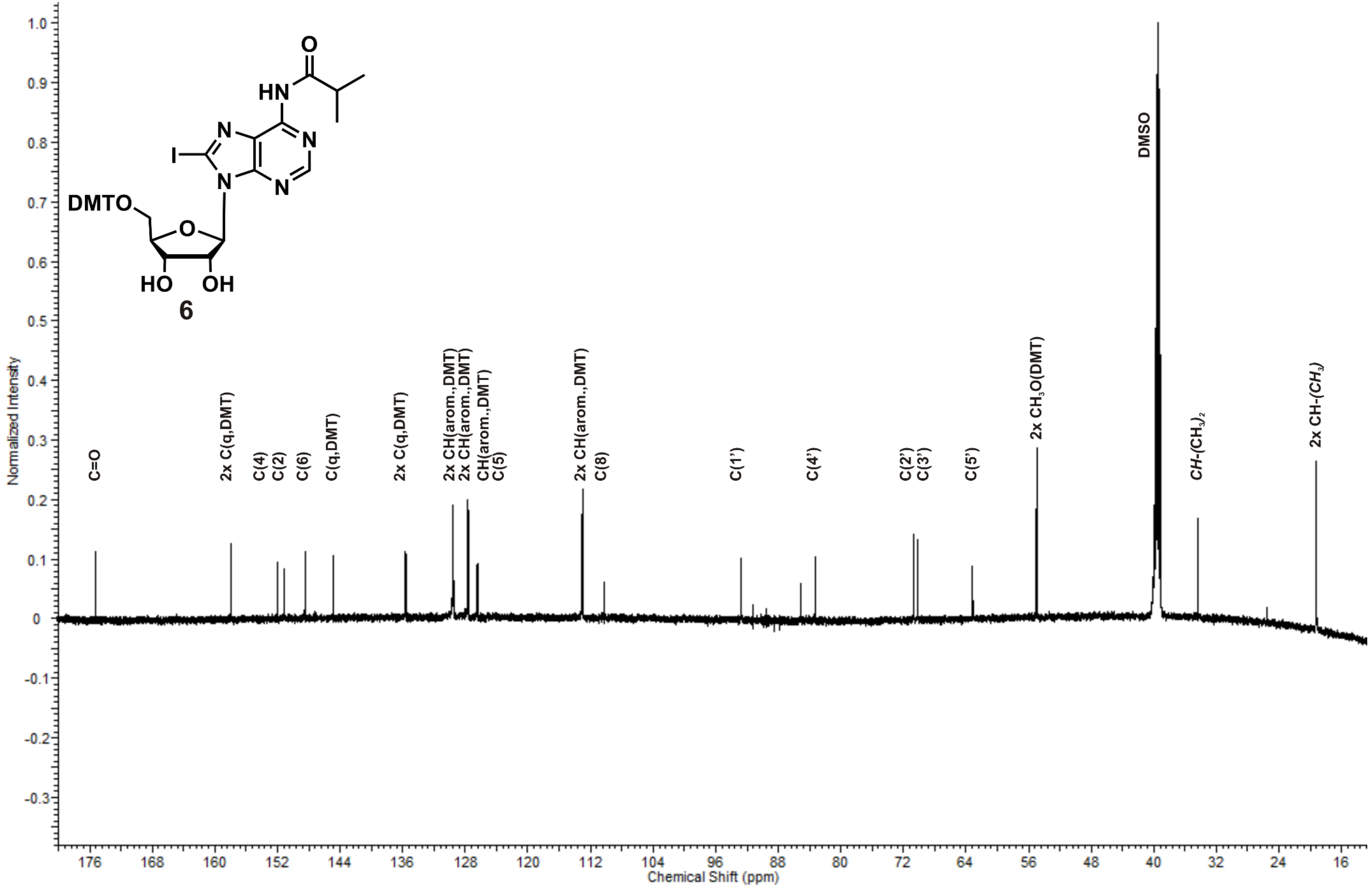
DMTO
HO
OH
O
N
HN
I
6
C=O
2x C(q,DMT)
C(4)
C(2)
C(6)
C(q,DMT)
2x C(q,DMT)
2x CH(arom.,DMT)
2x CH(arom.,DMT)
CH(arom.,DMT)
C(5)
2x CH(arom.,DMT)
C(8)
C(1')
C(4')
C(2')
C(3')
C(5')
2x CH3O(DMT)
DMSO
CH-(CH3)2
2x CH-(CH3)
Normalized Intensity
Chemical Shift (ppm)

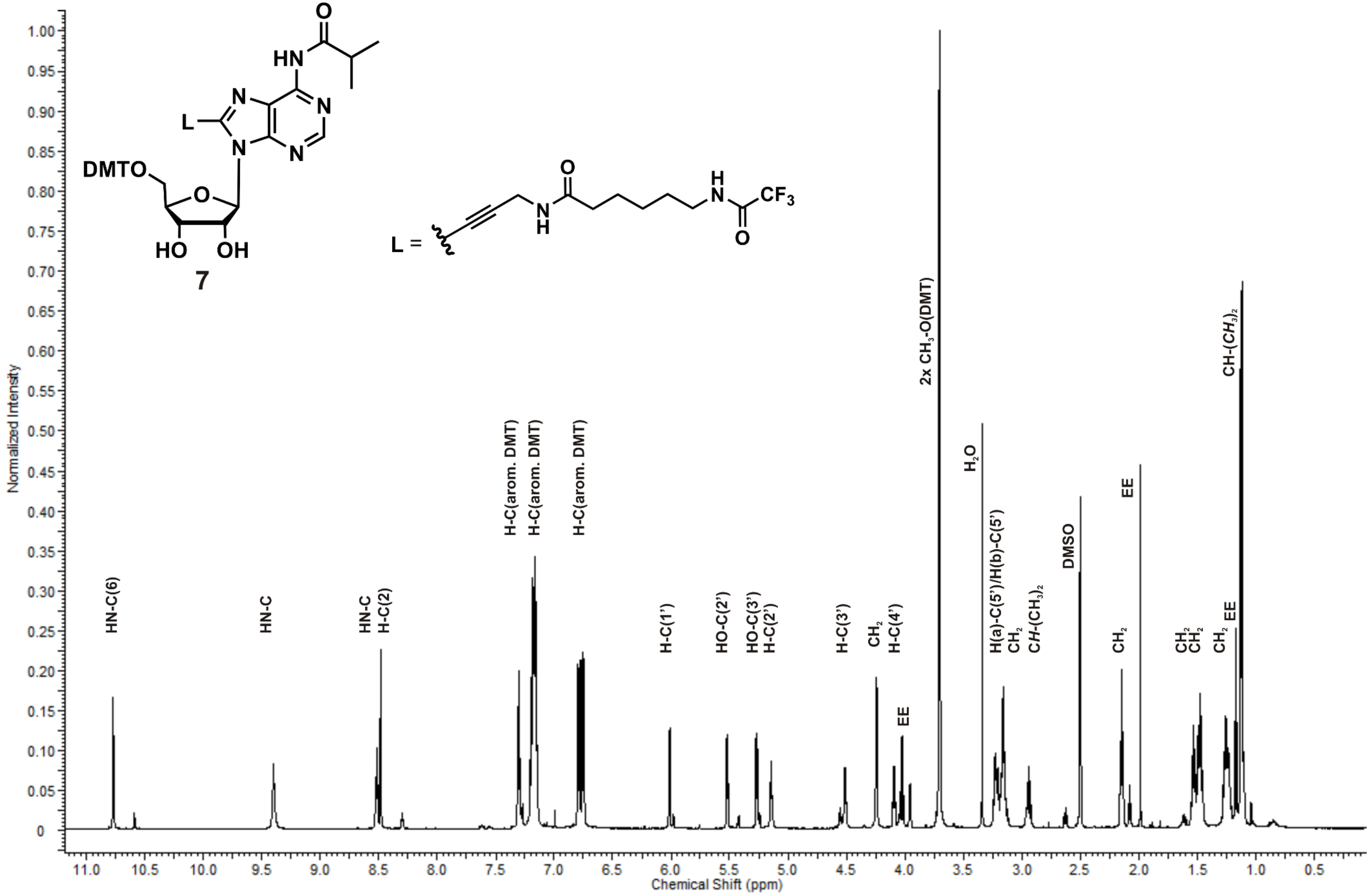

DMTO
HO
OH
7
L =
HN-C(6)
HN-C
HN-C
H-C(2)
H-C(arom. DMT)
H-C(arom. DMT)
H-C(arom. DMT)
H-C(1')
HO-C(2')
HO-C(3')
H-C(2')
H-C(3')
CH2
H-C(4')
EE
2x CH3-O(DMT)
H2O
H(a)-C(5')/H(b)-C(5')
CH2
CH-(CH3)2
DMSO
CH2
EE
CH2
CH2
CH2
EE
CH-(CH3)2
Normalized Intensity
Chemical Shift (ppm)

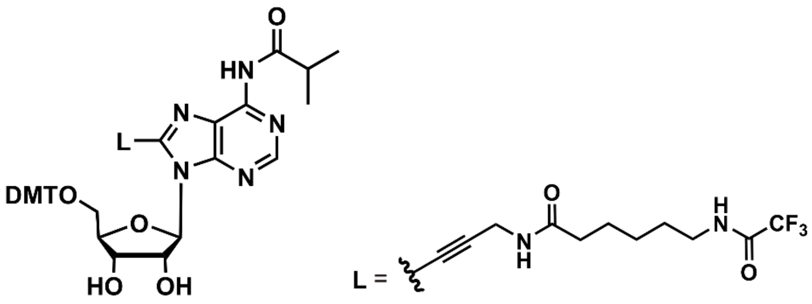
O
HN
N
N
L
N
N
DMTO
O
HO
OH
L =
O
N
H
H
N
CF3
O

7

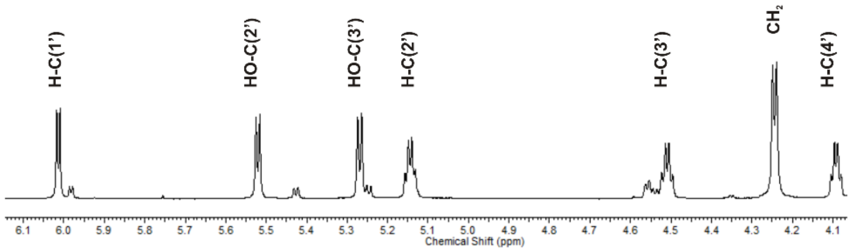
H-C(1')
HO-C(2')
HO-C(3')
H-C(2')
H-C(3')
CH2
H-C(4')
Chemical Shift (ppm)

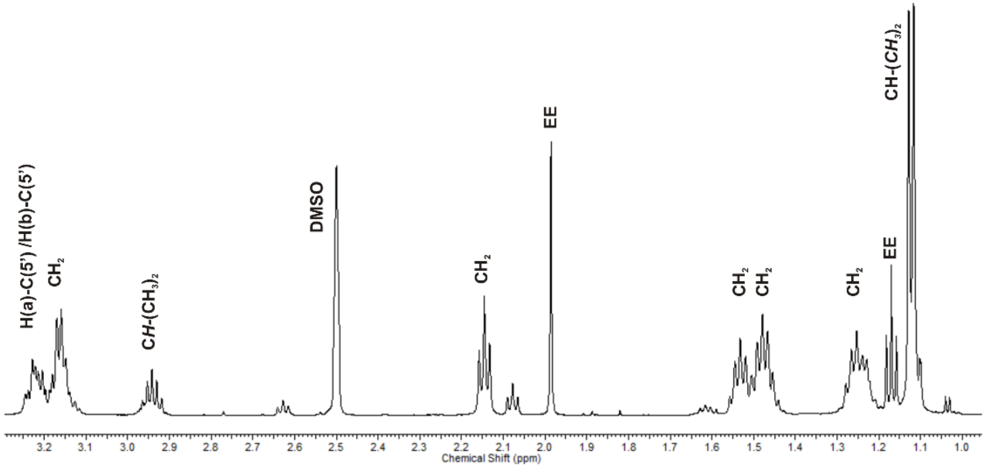
H(a)-C(5') /H(b)-C(5')
CH2
CH-(CH3)2
DMSO
CH2
EE
CH2
CH2
CH2
EE
CH-(CH3)2
Chemical Shift (ppm)

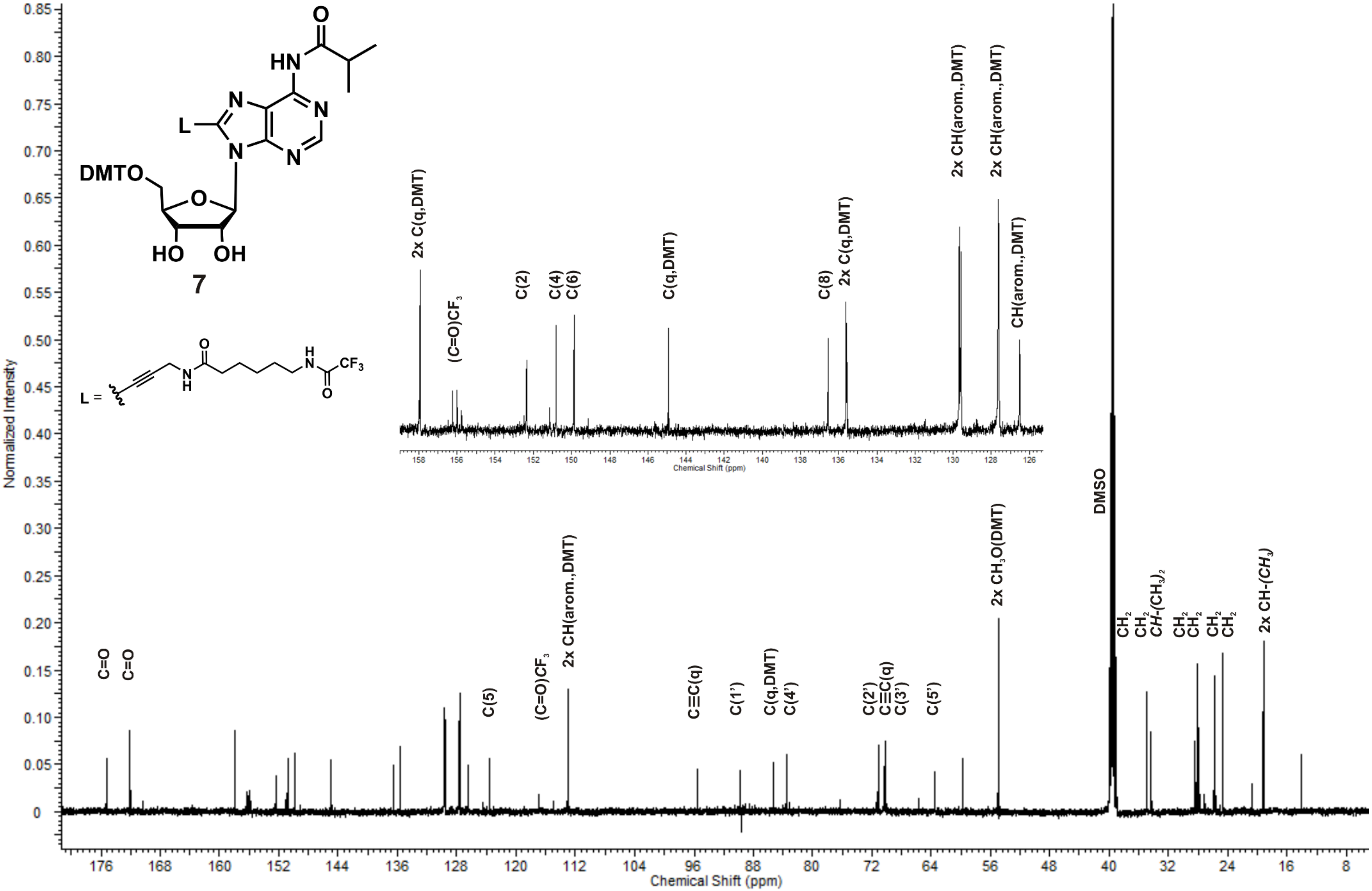

DMTO
HO
OH
7
L =
CF3
DMSO
2x CH3O(DMT)
2x CH-(CH3)
C=O
C=O
C(5)
(C=O)CF3
2x CH(arom.,DMT)
C≡C(q)
C(1')
C(q,DMT)
C(4')
C(2')
C≡C(q)
C(3')
C(5')
CH2
CH2
CH-(CH3)2
CH2
CH2
CH2
CH2
2x C(q,DMT)
(C=O)CF3
C(2)
C(4)
C(6)
C(q,DMT)
C(8)
2x C(q,DMT)
2x CH(arom.,DMT)
2x CH(arom.,DMT)
CH(arom.,DMT)
Chemical Shift (ppm)
Normalized Intensity

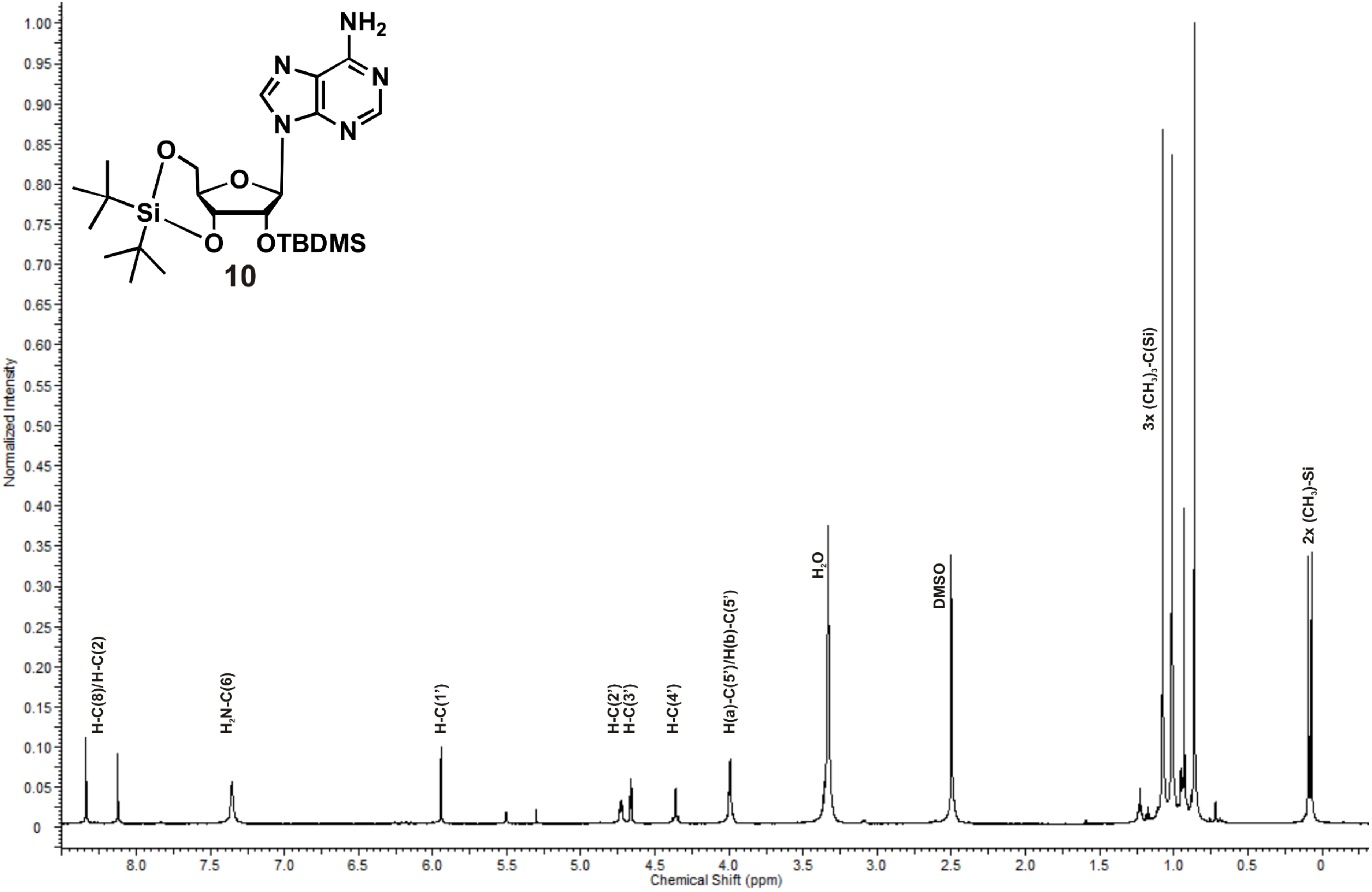

NH2
N
N
N
N
O
O
Si
O
OTBDMS
10
Normalized Intensity
Chemical Shift (ppm)
H-C(8)/H-C(2)
H2N-C(6)
H-C(1')
H-C(2')
H-C(3')
H-C(4')
H(a)-C(5')/H(b)-C(5')
H2O
DMSO
3x (CH3)3-C(Si)
2x (CH3)-Si

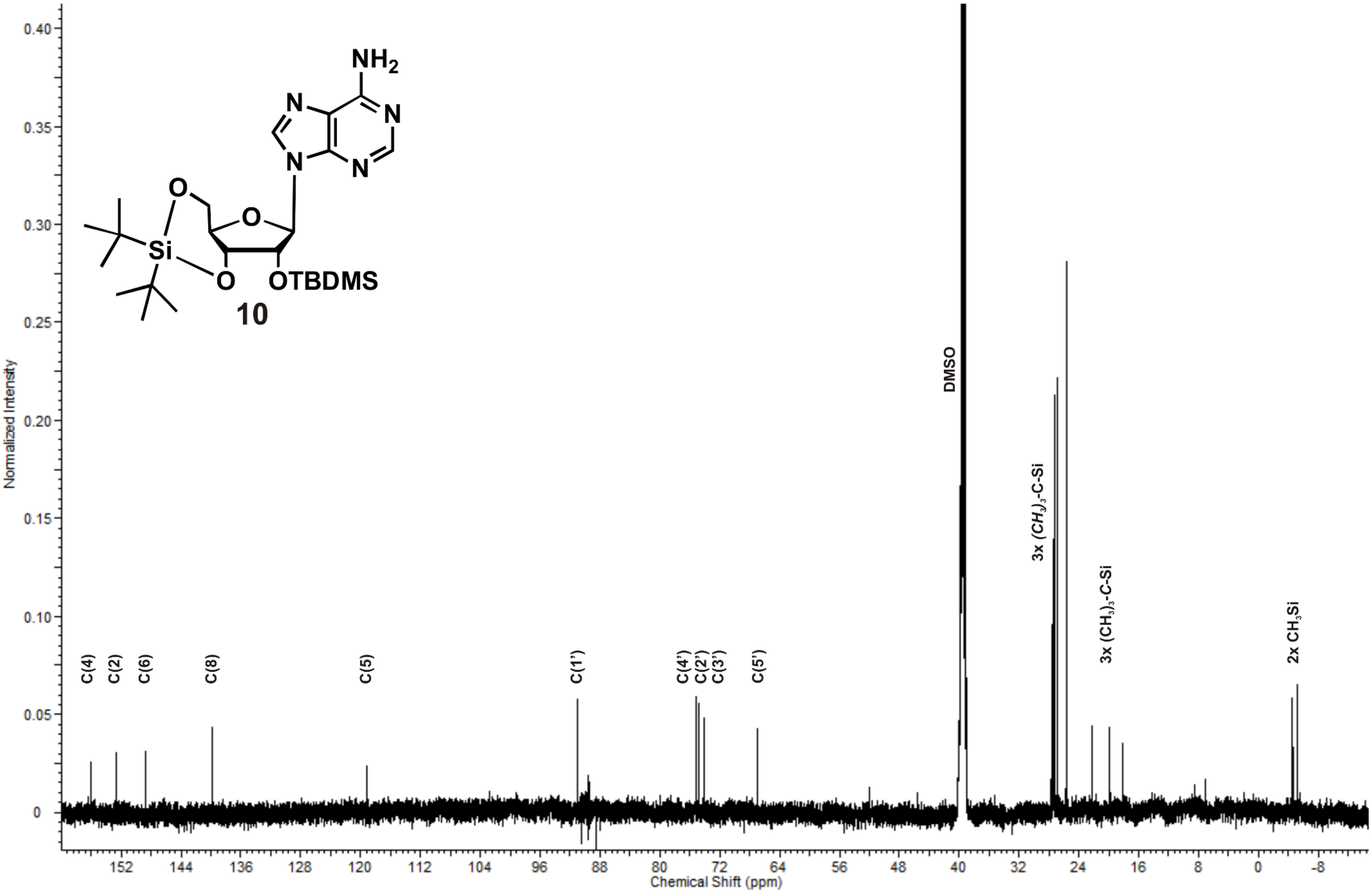

NH2
N
N
N
N
O
O
Si
O
OTBDMS
10
Normalized Intensity
0.40
0.35
0.30
0.25
0.20
0.15
0.10
0.05
0
DMSO
3x (CH3)3-C-Si
3x (CH3)3-C-Si
2x CH3Si
C(4)
C(2)
C(6)
C(8)
C(5)
C(1')
C(4')
C(2')
C(3')
C(5')
152
144
136
128
120
112
104
96
88
80
72
64
56
48
40
32
24
16
8
0
-8
Chemical Shift (ppm)

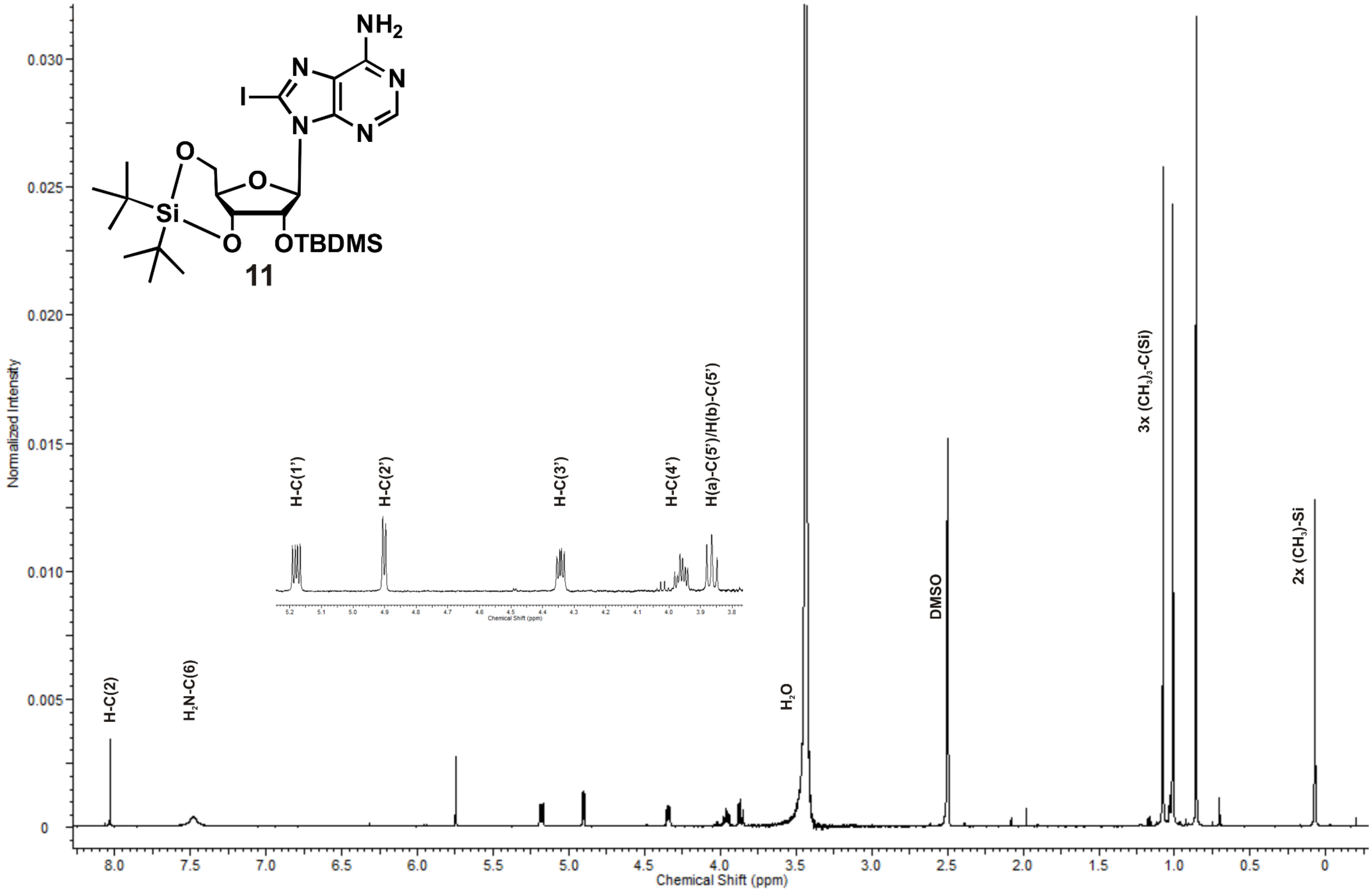
NH2
N
N
I
N
N
O
O
Si
O
O
OTBDMS
11
H-C(1')
H-C(2')
H-C(3')
H-C(4')
H(a)-C(5')/H(b)-C(5')
Chemical Shift (ppm)
H-C(2)
H2N-C(6)
H2O
DMSO
3x (CH3)3-C(Si)
2x (CH3)-Si
Normalized Intensity
Chemical Shift (ppm)

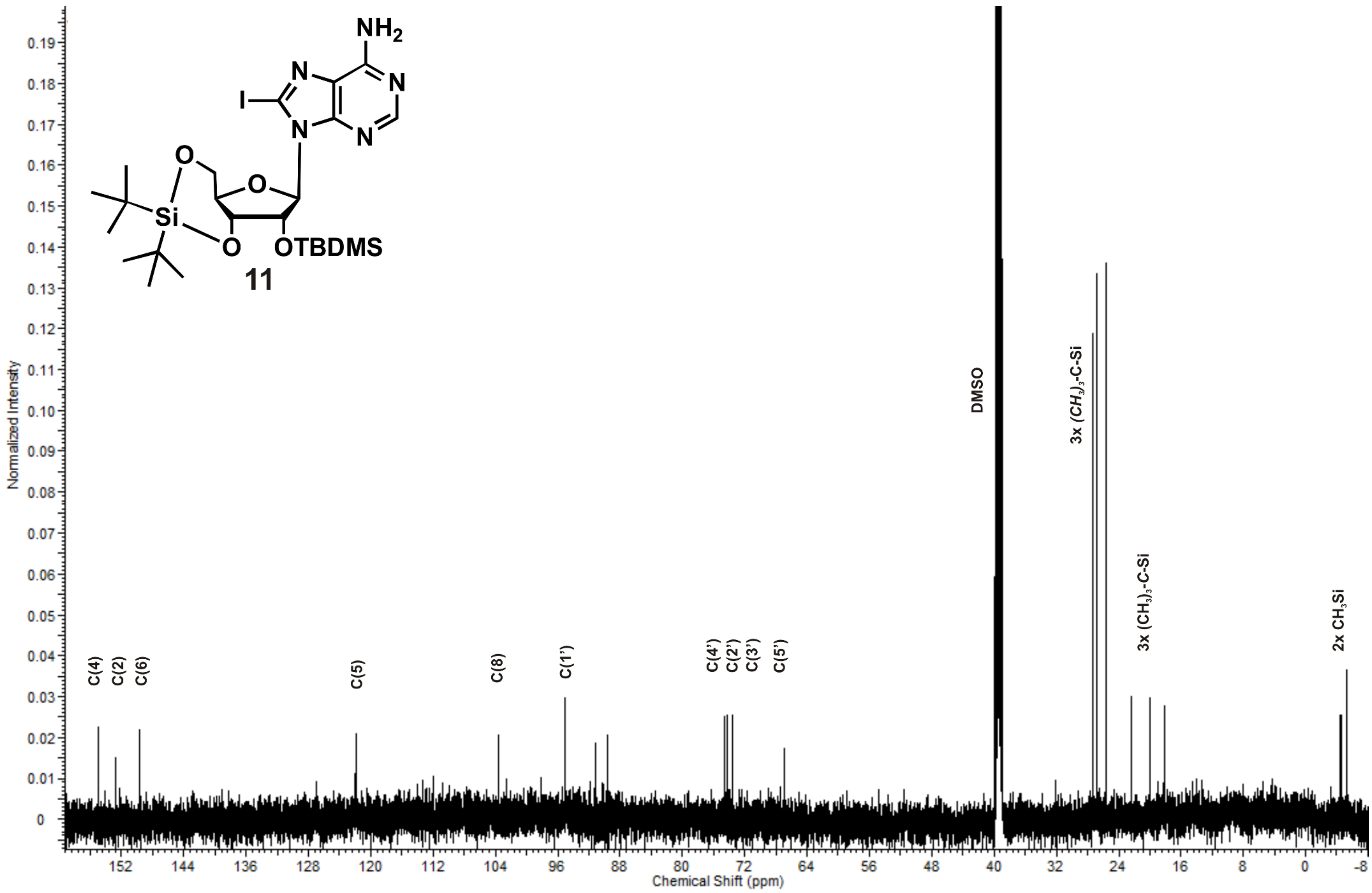

NH2
N
N
I
N
N
O
O
Si
O
OTBDMS
11
O
Normalized Intensity
Chemical Shift (ppm)
C(4)
C(2)
C(6)
C(5)
C(8)
C(1')
C(4')
C(2')
C(3')
C(5')
DMSO
3x (CH3)3-C-Si
3x (CH3)3-C-Si
2x CH3Si

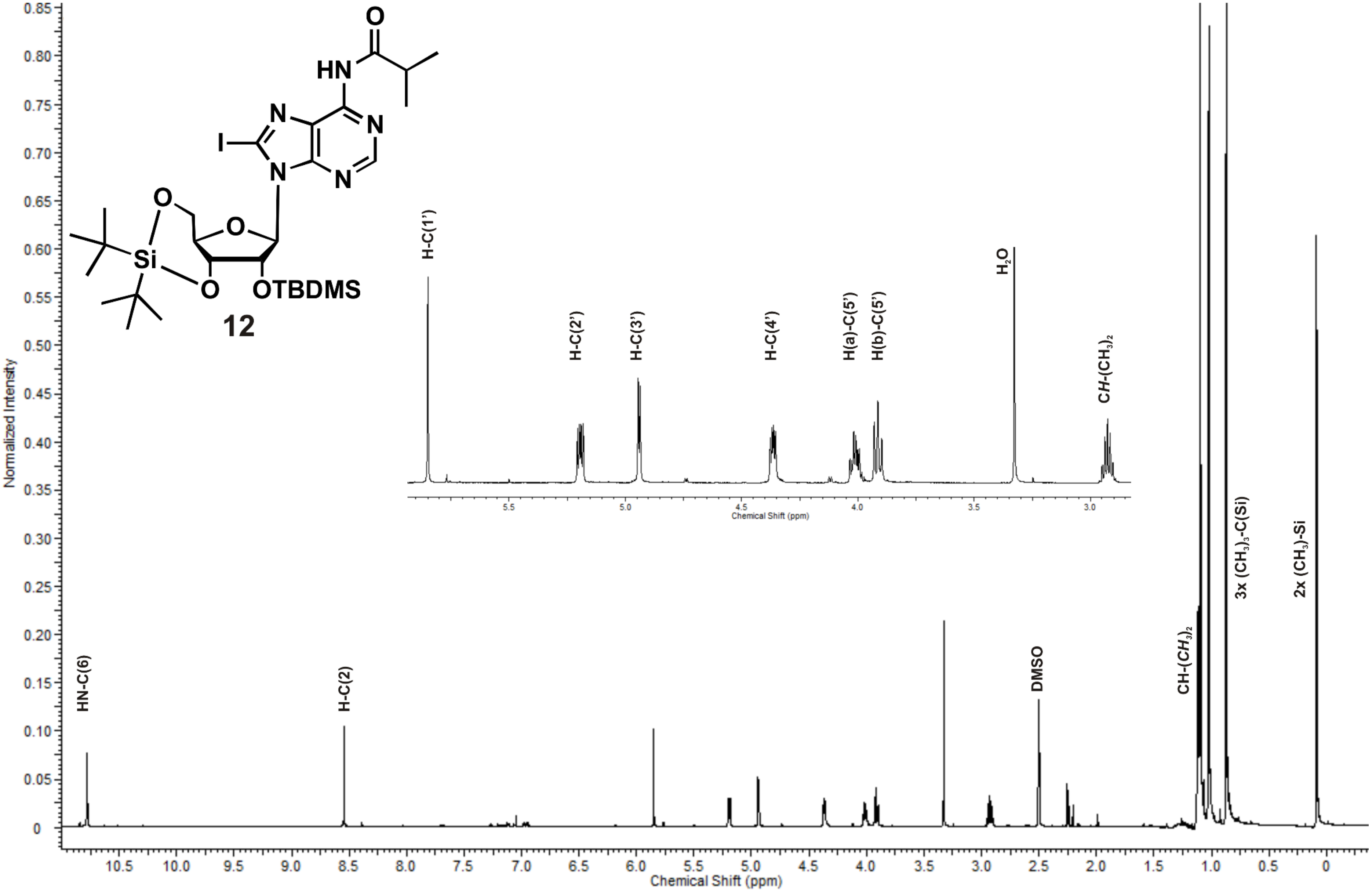

12
HN-C(6)
H-C(2)
DMSO
CH-(CH3)2
3x (CH3)3-C(Si)
2x (CH3)-Si
H-C(1')
H-C(2')
H-C(3')
H-C(4')
H(a)-C(5')
H(b)-C(5')
H2O
CH-(CH3)2
Normalized Intensity
Chemical Shift (ppm)

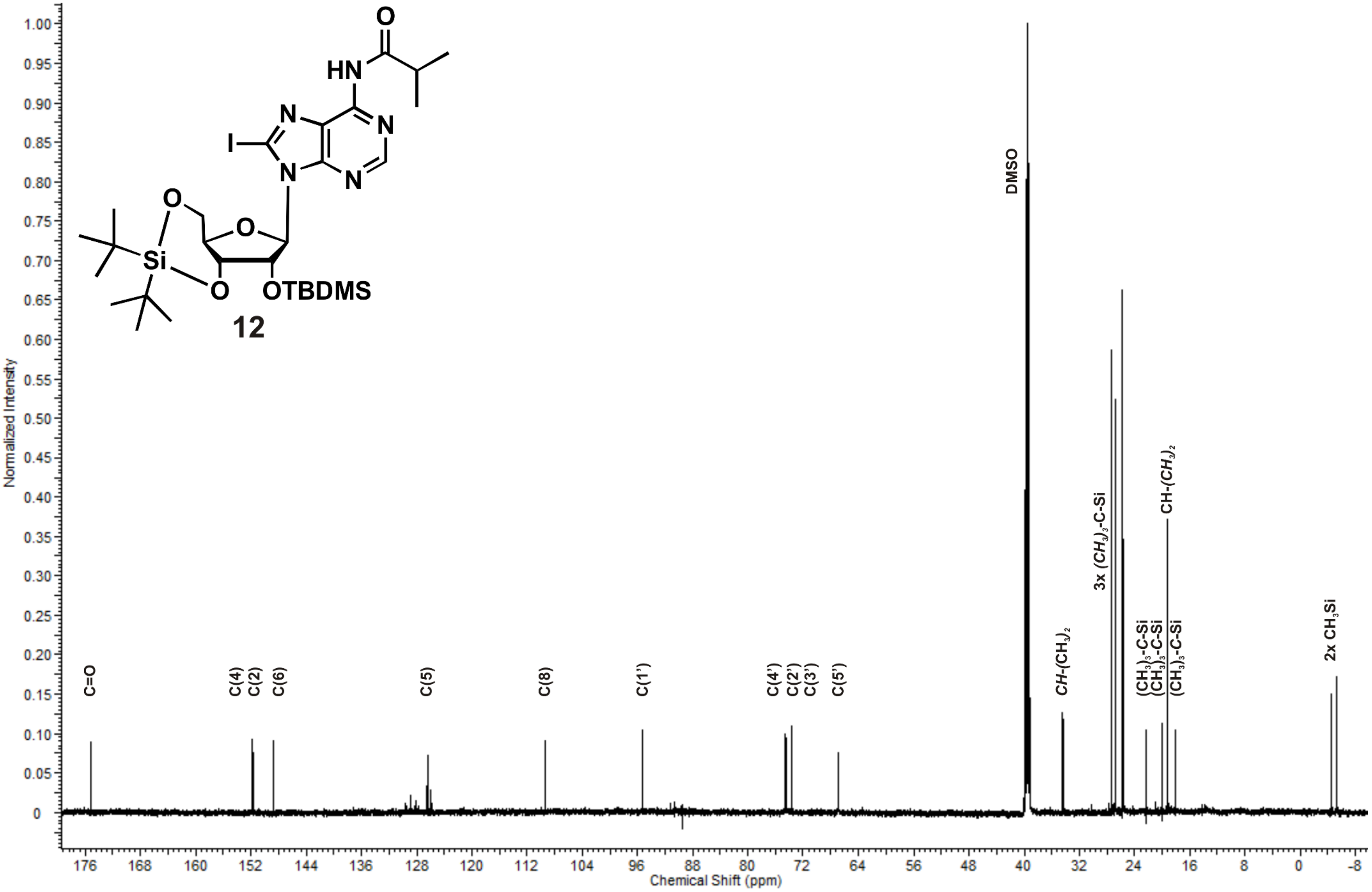

O
HN
N
N
I
N
N
O
O
Si
O
O
OTBDMS
12
DMSO
C=O
C(4)
C(2)
C(6)
C(5)
C(8)
C(1')
C(4')
C(2')
C(3')
C(5')
CH-(CH3)2
3x (CH3)3-C-Si
(CH3)3-C-Si
(CH3)3-C-Si
CH-(CH3)2
(CH3)3-C-Si
2x CH3Si
Normalized Intensity
Chemical Shift (ppm)

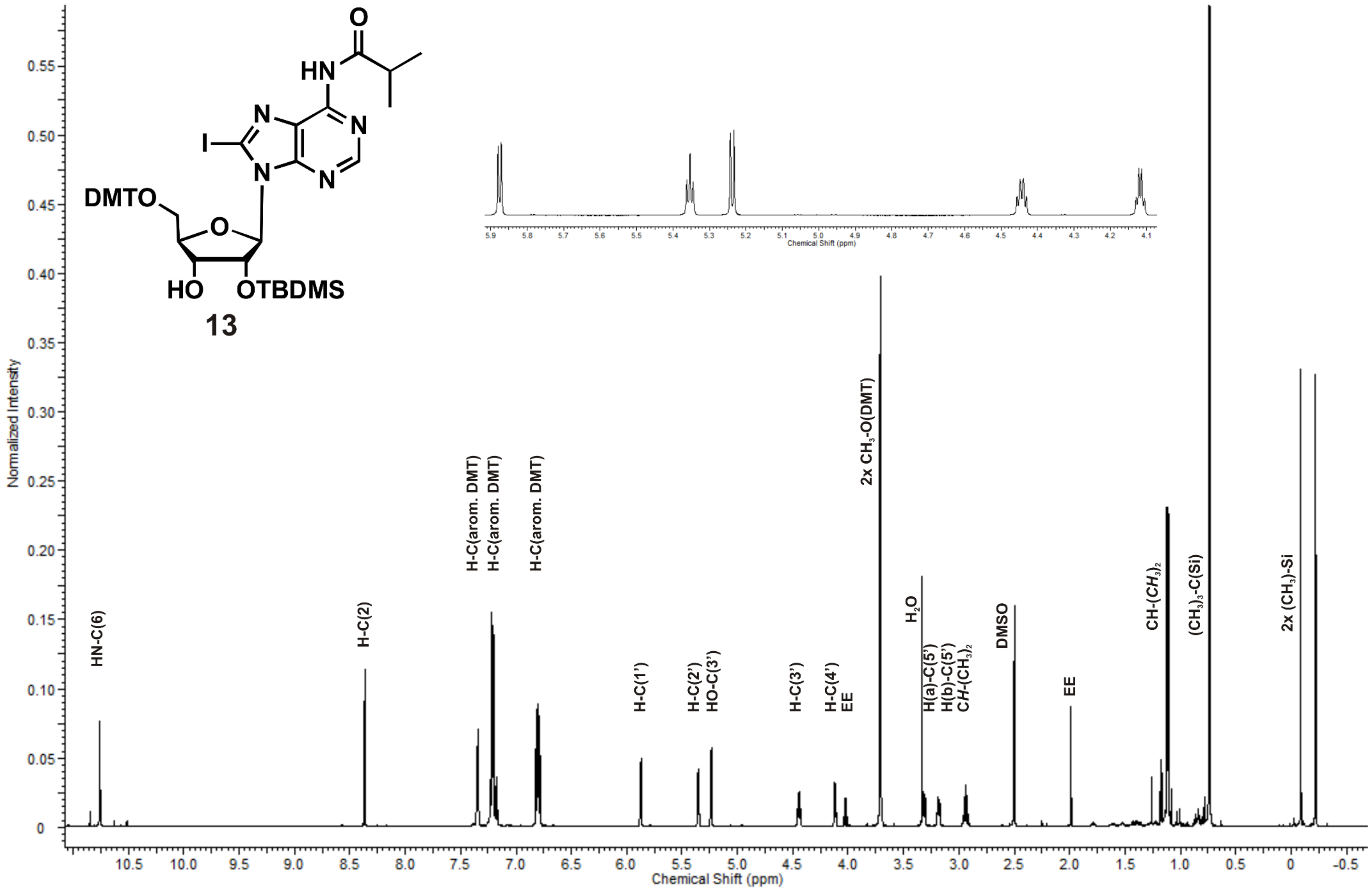
DMTO
HO
OTBDMS
13
HN
O
N
I
HN-C(6)
H-C(2)
H-C(arom. DMT)
H-C(arom. DMT)
H-C(arom. DMT)
H-C(1')
H-C(2')
HO-C(3')
H-C(3')
H-C(4')
EE
2x CH3-O(DMT)
H2O
H(a)-C(5')
H(b)-C(5')
CH-(CH3)2
DMSO
EE
CH-(CH3)2
(CH3)3-C(Si)
2x (CH3)-Si
Normalized Intensity
Chemical Shift (ppm)

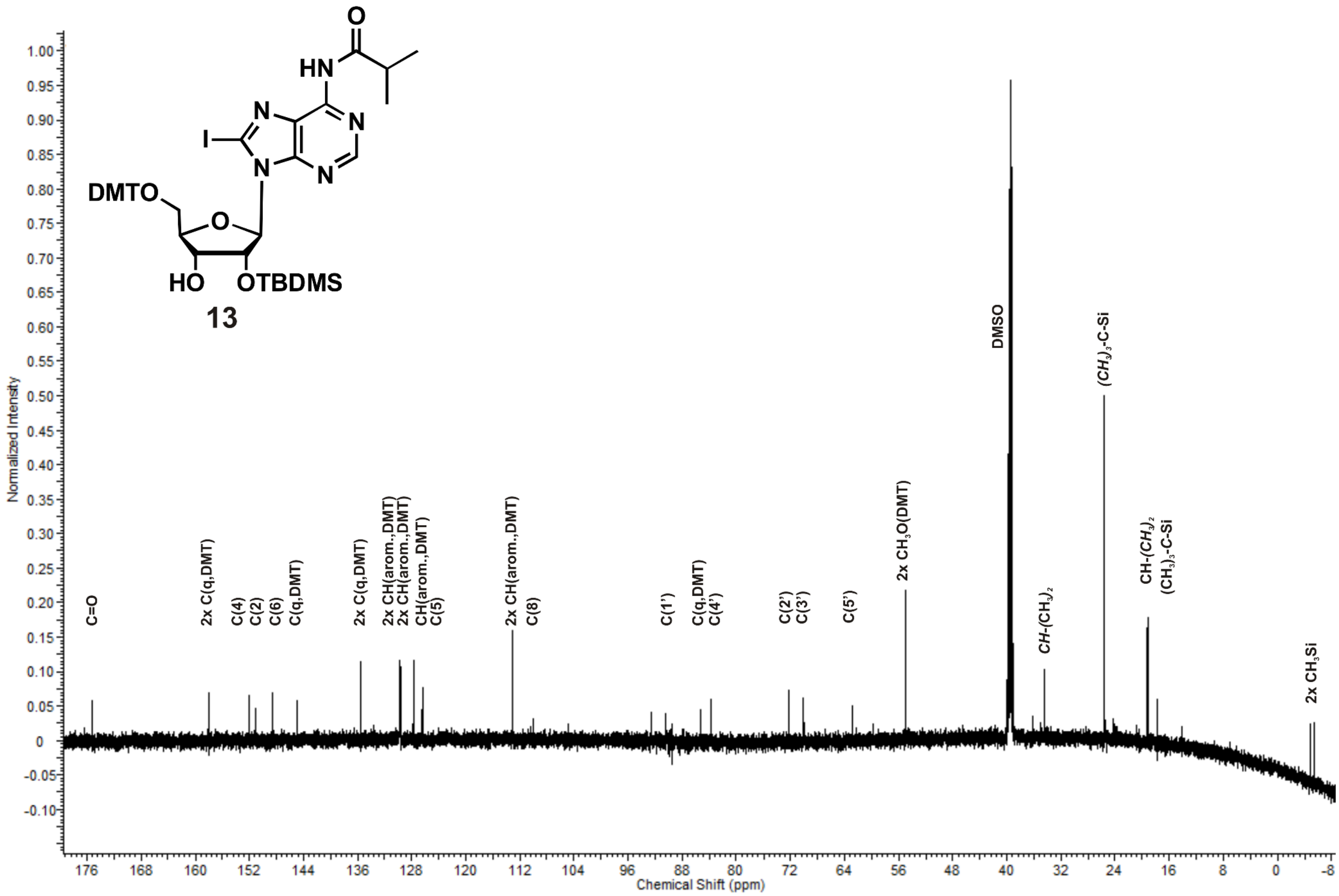

13
DMSO
C=O
2x C(q,DMT)
C(4)
C(2)
C(6)
C(q,DMT)
2x C(q,DMT)
2x CH(arom.,DMT)
2x CH(arom.,DMT)
CH(arom.,DMT)
C(5)
2x CH(arom.,DMT)
C(8)
C(1')
C(q,DMT)
C(4')
C(2')
C(3')
C(5')
2x CH3O(DMT)
CH-(CH3)2
(CH3)3-C-Si
CH-(CH3)2
(CH3)3-C-Si
2x CH3Si
Normalized Intensity
Chemical Shift (ppm)

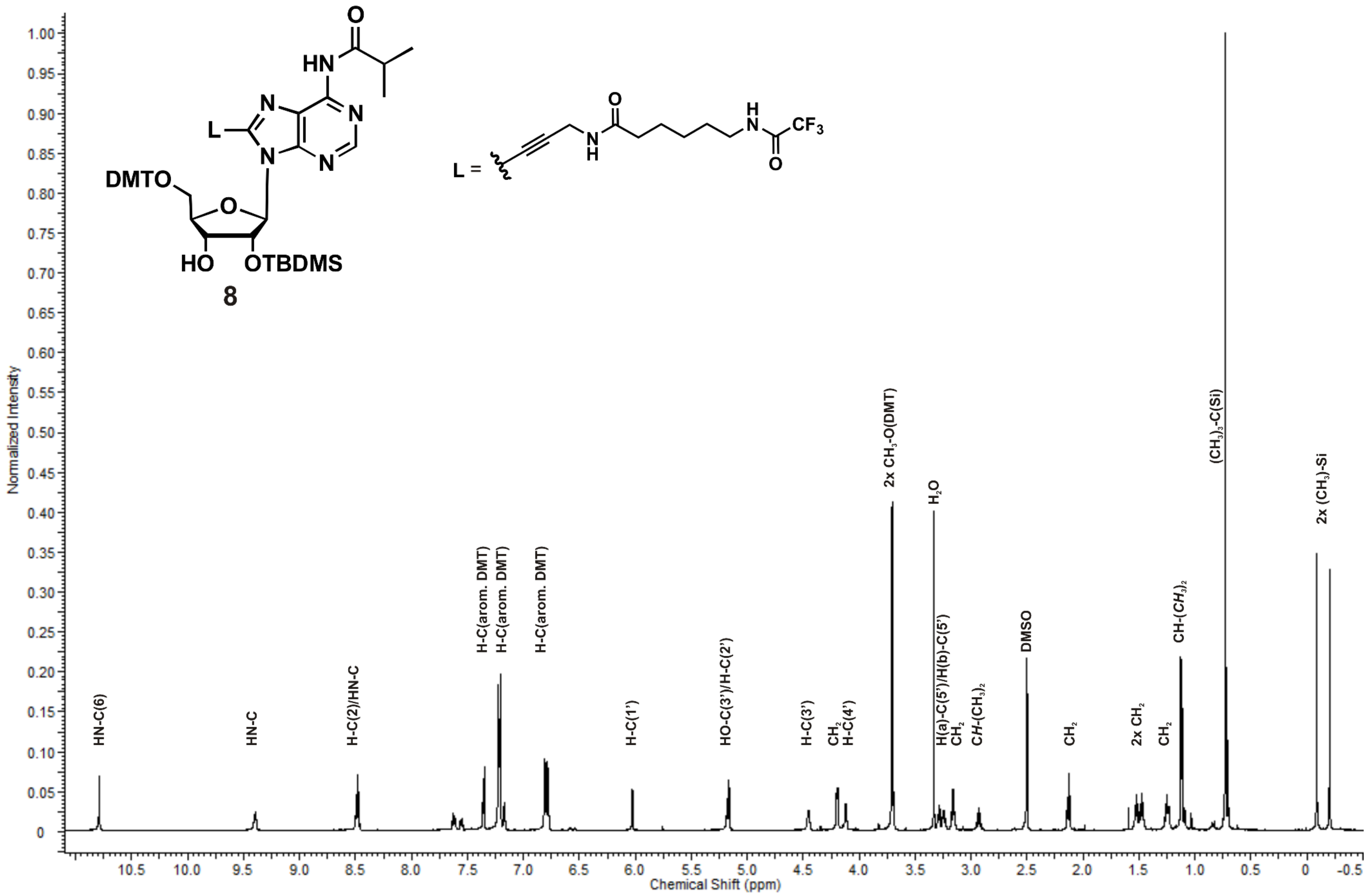

DMTO
HO
OTBDMS
8
HN
O
N
L
L =
CF3
Normalized Intensity
Chemical Shift (ppm)
HN-C(6)
HN-C
H-C(2)/HN-C
H-C(arom. DMT)
H-C(arom. DMT)
H-C(arom. DMT)
H-C(1')
HO-C(3')/H-C(2')
H-C(3')
CH2
H-C(4')
2x CH3-O(DMT)
H2O
H(a)-C(5')/H(b)-C(5')
CH2
CH-(CH3)2
DMSO
CH2
2x CH2
CH2
CH-(CH3)2
(CH3)3-C(Si)
2x (CH3)-Si

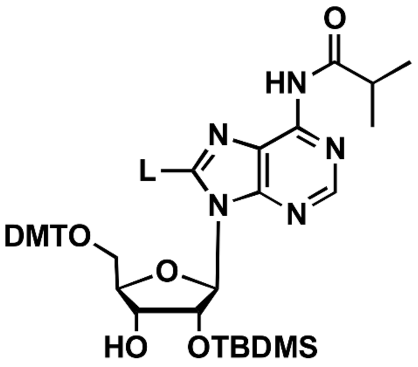
DMTO
HO
OTBDMS
L
HN
N
O

8

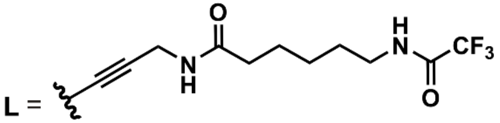
L =
CF3

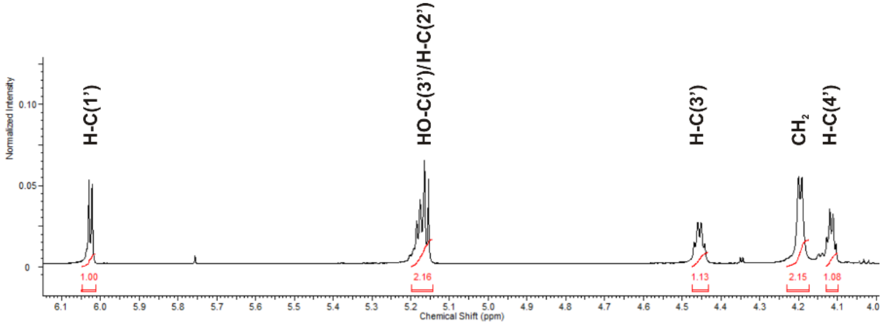
H-C(1')
HO-C(3')/H-C(2')
H-C(3')
CH2
H-C(4')
Normalized Intensity
Chemical Shift (ppm)

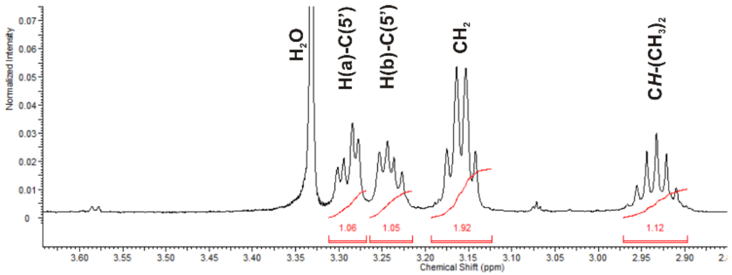
H2O
H(a)-C(5')
H(b)-C(5')
CH2
CH-(CH3)2
Normalized Intensity
Chemical Shift (ppm)

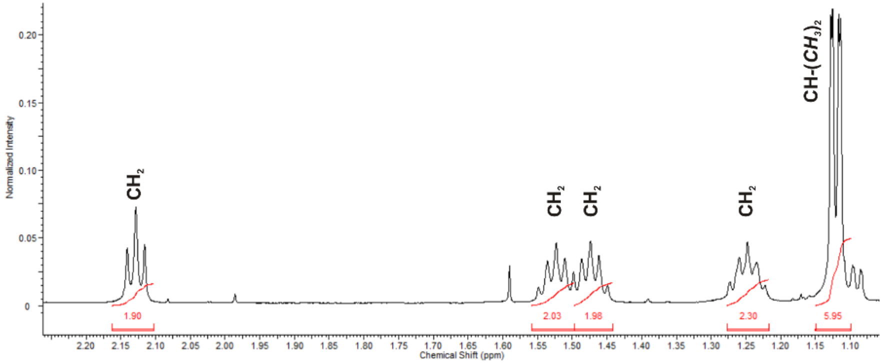
CH2
CH2
CH2
CH2
CH-(CH3)2
Normalized Intensity
Chemical Shift (ppm)

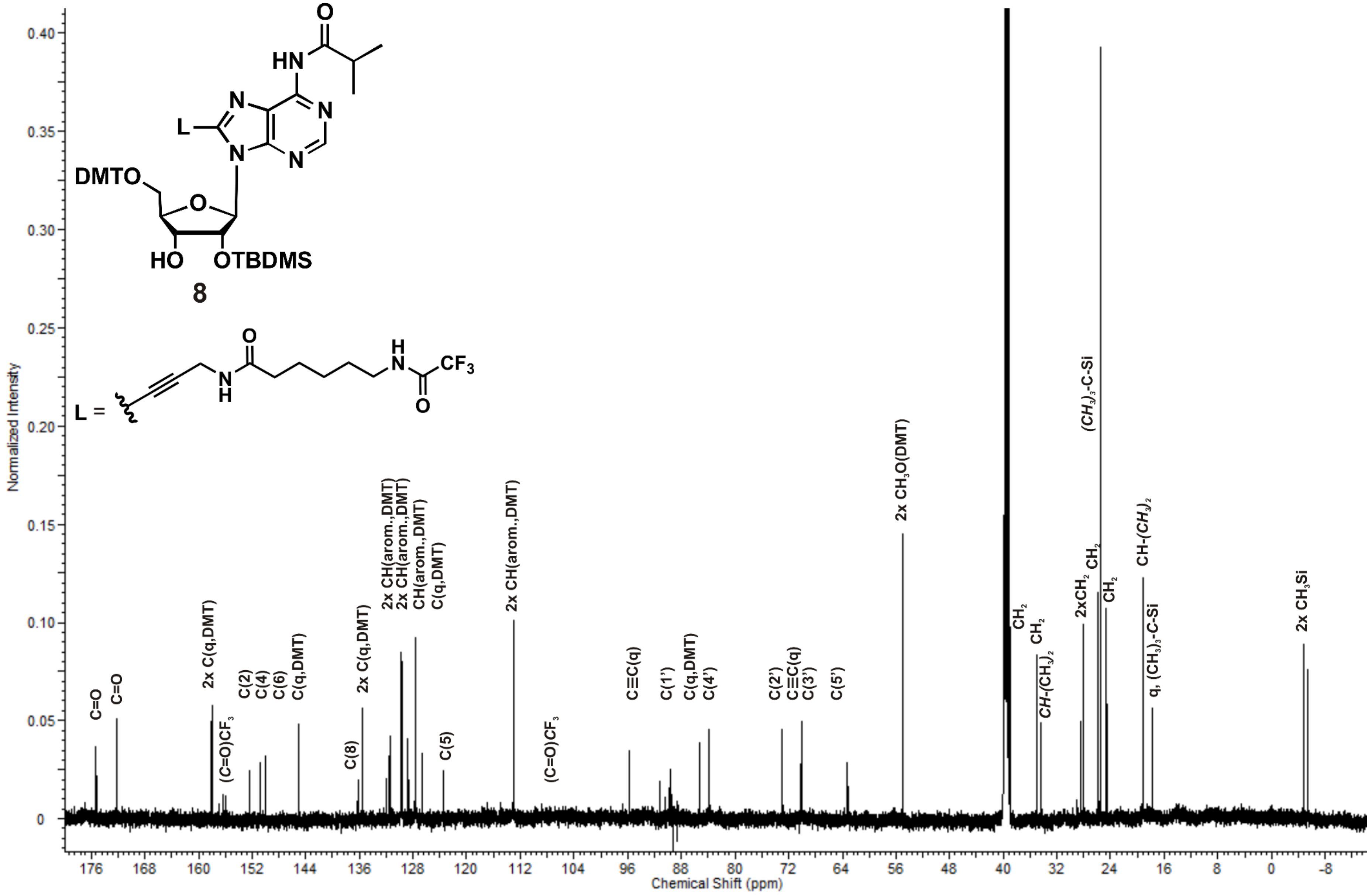

8
L =
Normalized Intensity
Chemical Shift (ppm)
C=O
C=O
2x C(q,DMT)
(C=O)CF3
C(2)
C(4)
C(6)
C(q,DMT)
C(8)
2x C(q,DMT)
2x CH(arom.,DMT)
2x CH(arom.,DMT)
CH(arom.,DMT)
C(q,DMT)
C(5)
2x CH(arom.,DMT)
(C=O)CF3
C≡C(q)
C(1')
C(q,DMT)
C(4')
C(2')
C≡C(q)
C(3')
C(5')
2x CH3O(DMT)
CH2
CH2
CH-(CH3)2
2xCH2
CH2
(CH3)3-C-Si
CH2
CH-(CH3)2
q, (CH3)3-C-Si
2x CH3Si
